# Effect of a specific food intervention with Tamogitake mushroom, Moringa leaves, or rice bran on intestinal microbiota and cognitive function in elderly Japanese

**DOI:** 10.3389/fnut.2025.1585111

**Published:** 2025-07-21

**Authors:** Kouta Hatayama, Kanako Kono, Kana Okuma, Hiroaki Masuyama

**Affiliations:** Symbiosis Solutions, Inc., Tokyo, Japan

**Keywords:** intestinal microbiota, cognitive function, Japan, mild cognitive impairment, dementia, Tamogitake, Moringa, rice bran

## Abstract

**Introduction:**

The large number of elderly patients with dementia and mild cognitive impairment with cognitive decline in Japan has become a social problem. In this study, a food intervention study was conducted to test whether a food intervention approach targeting intestinal microbiota can improve cognitive function in elderly Japanese individuals.

**Methods:**

Japanese participants (144 males and 87 females) aged 60–79 years were assigned to one of the test food groups: Tamogitake mushroom (*Pleurotus cornucopiae* var. *citrinopileatus*), *Moringa oleifera* Lam. leaves, and rice bran for each sex, respectively. After 4 weeks of pre-observation, each group consumed the test foods for 8 weeks; cognitive function and intestinal microbiota tests were performed after each 4-week period. The intestinal microbiota of each participant was determined by 16S rRNA gene amplicon sequencing.

**Results:**

The participants were divided into responders (improved cognitive function) and non-responders (no improvement) within each sex group. Responders exhibited variations in intestinal bacteria belonging to specific taxa, including *Agathobaculum*, *Anaerobutyricum*, *Blautia*, *Faecalibacterium*, *Parabacteroides*, and *Phascolarctobacterium*, compared to non-responders, indicating that cognitive function may be improved by changes in specific intestinal bacteria with food intake. However, improvements in cognitive function would require consuming foods suitable for the composition of the intestinal microbiota.

**Conclusion:**

Food intervention approaches targeting the intestinal microbiota could lead to innovative solutions for improving cognitive function in the elderly.

## 1 Introduction

In many countries around the world, the population is aging. In Japan, 29.1% of the population was reported to be 65 years old or older in 2023 (1). In addition, the number of elderly patients with dementia and mild cognitive impairment (MCI) in Japan was estimated to be 4,443,000 and 5,585,000, respectively, in 2022, placing a major health, welfare, and economic burden on the society (1, 2).

There is no definitive cure for dementia, and recovery from the disease is difficult; thus, preventing its onset is important. MCI does not significantly interfere with daily life, but involves cognitive decline and is associated with a high risk of progressing to dementia (3–5). Unlike those with dementia, patients with MCI may return to normal cognitive function, and appropriate interventions for patients with MCI may improve cognitive function or inhibit progression to dementia (3, 6). Preventing dementia and MCI or at least preventing cognitive decline at the MCI stage before it progresses to dementia is one of the most important measures in an aging society.

Previously, we reported that the intestinal microbiota may play a significant role in the development and progression of MCI (7). It is hypothesized that the characteristic dysbiosis of intestinal microbiota in MCI leads to dysregulation of the intestinal microbiota, increased permeability of the intestinal and blood–brain barriers, and increased chronic neuroinflammation, which persists for long periods (over 10–20 years), ultimately leading to cognitive decline. In addition, we constructed MCI risk estimation models for both sexes based on intestinal microbiota data, and these models could well discriminate between MCI and healthy control groups (7). Since the composition of intestinal microbiota is greatly affected by diet, dietary changes may help prevent or improve dysbiosis of intestinal microbiota associated with MCI. Based on these, early detection of MCI and improvement of the intestinal microbiota through diet-related intervention approaches are expected to lead to innovative strategies to delay or prevent the progression from MCI to dementia.

Although few studies have analyzed changes in the intestinal microbiota and cognitive function in older adults through dietary intervention, adherence to the Mediterranean diet or modified Mediterranean-ketogenic diet has been reported to lead to changes in specific intestinal bacteria and improving cognitive function in older adults (8–10). However, changing and adhering to these dietary patterns may be difficult for Japanese people, who have a different food culture. In contrast, food intervention is easier for elderly Japanese people to adopt. Nevertheless, while various foods such as vegetables, nuts, berries, extra-virgin olive oil, coffee, cocoa, tea, Tamogitake (*Pleurotus cornucopiae* var. *citrinopileatus*), Moringa (*Moringa oleifera* Lam.) leaves, and rice bran have been studied for their effects on cognitive function, there is not yet strong evidence to support these effects (11–16). Furthermore, we have not found any studies that analyzed the association among food intervention, cognitive function, and intestinal microbiota in older adults. Therefore, whether these foods can improve the intestinal microbiota dysbiosis associated with MCI in Japanese elderly people was unclear.

Therefore, we planned a human food intervention study as the next stage to verify whether a food intervention approach targeting intestinal microbiota can improve cognitive function in elderly Japanese people with suspected MCI. Tamogitake, Moringa leaves, and rice bran were selected as the test foods because of their potential to improve cognitive function. Tamogitake is an edible mushroom containing high levels of L-ergothioneine, which is reported to improve memory and learning abilities (12, 13). Moringa leaves are nutritionally balanced and contain a variety of antioxidant compounds, and their extracts have been reported to be beneficial against Alzheimer’s disease (14, 15). Rice bran potentially reduces neuroinflammation associated with Alzheimer’s disease and helps improve cognition and memory (16). Here, one of these three test foods was continuously consumed for 8 weeks by Japanese males and females aged 60–79 years who did not have high cognitive function, and changes in cognitive function and intestinal microbiota were observed.

## 2 Materials and methods

### 2.1 Mini-mental state examination

The Japanese version of mini-mental state examination (MMSE-J; Nihon Bunka Kagakusha Co., Ltd., Tokyo, Japan) was conducted (17). The MMSE is widely used as a simple screening test for dementia and MCI, as well as a measure of general cognitive function. The total MMSE score ranges from 0 to 30, with lower scores indicating lower cognitive function. A score of 28 or higher indicates normal cognitive function, 24–27 indicates suspected MCI, and 23 or less indicates suspected dementia (18).

### 2.2 Cognitrax test

Cognitrax (Health Solution, Inc., Tokyo, Japan) is a computer-based cognitive function test developed as a Japanese version of the CNS Vital Signs (CNS Vital Signs LLC, Morrisville, NC, USA) (19). The test battery includes seven tests: verbal memory test, visual memory test, finger tapping test, symbol digit cording test, Stroop test, shifting attention test, and continuous performance test. The neurocognitive index (NCI) and scores for 11 domains (composite memory, verbal memory, visual memory, psychomotor speed, reaction time, complex attention, cognitive flexibility, processing speed, executive function, simple attention, and motor speed) were calculated by combining the results of individual tests. The NCI and scores for the 11 domains were age-adjusted and standardized by setting a mean score of 100 and standard deviation of 15. The NCI and each score were judged as follows: >110, high function; 90–110, standard function; 80–89, slight impairment; 70–79, possible mild impairment; and <70, possible impairment.

### 2.3 Test and analysis of intestinal microbiota

Stool sample collection, DNA extraction, and 16S rRNA gene sequence analysis (variable regions V1 to V3) using the MiSeq system (Illumina, San Diego, CA, USA) were performed according to the method described by Hatayama et al. (7). The taxonomic assignment of amplicon sequence variants (ASVs) was determined using the Ribosomal Database Project training set v.18 (20).^1^ The transformation of abundance into a central log-ratio (CLR) for each taxon was performed using R v. 4.2.0 ALDEx2v. 1.28.1 package (21). To visualize β-diversity, non-metric multidimensional scaling (NMDS) based on the Bray–Curtis index was used. The metaMDS function in R v. 4.2.0 vegan v. 2.6-4 package (22) was used for NMDS.

### 2.4 Participant recruitment

A total of 1,229 Japanese males and females aged 60–79 years were recruited through a medical volunteer recruitment company (SOUKEN, Tokyo, Japan). The inclusion criteria were as follows: being concerned about one’s own memory loss, ability to defecate normally and having a frequency of defecation of at least once every 3 days, and being a Japanese and living in Japan since birth. Written informed consent was obtained from all the participants. The study was conducted according to the guidelines of the Declaration of Helsinki and approved by the Institutional Review Board of Shiba Palace Clinic (Tokyo, Japan) (approval number and date: 152772-35569, 5 October 2023).

The following 10 criteria were used to exclude participants: (1) showing allergic symptoms to the test foods (Tamogitake, Moringa, or rice bran), (2) participating in other clinical trials, (3) judged by the study staff to have some kind of a problem (e.g., eligibility of participants and compliance with the study protocol), (4) regular intake of test foods (Tamogitake, Moringa, or rice bran), (5) regular intake of health foods or supplements that claim to have effects such as lactic acid bacteria, oligosaccharides, or intestinal regulation, (6) use of antibiotics within the past month, (7) planning to make major changes to diet or lifestyle (exercise, smoking, employment, and living environment) during the study period, (8) planning to travel overseas during the study period, (9) being a heavy drinker (drinking alcohol equivalent to 360 ml or more of sake per day on more than half of the days in a week), and (10) being a heavy smoker (smoking more than 40 cigarettes per day).

### 2.5 Screening of participants and group assignment

**FIGURE 1:**
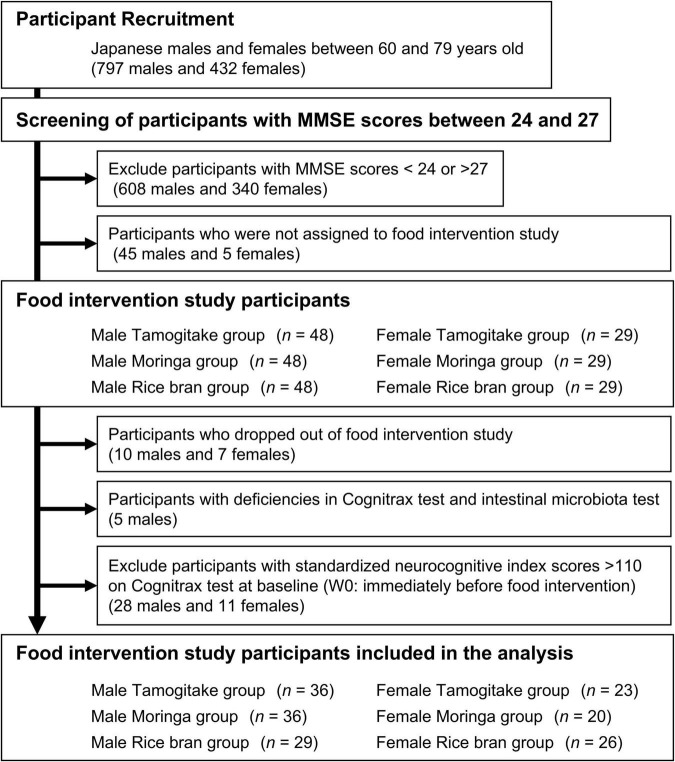
The process from recruiting participants for the food intervention study to selecting the subjects for analysis.

Among the participants, those who met the inclusion criteria and had a MMSE score between 24 or more and 27 or less (level of cognitive function suspected to be MCI) were screened (189 males and 92 females). One hundred forty-four males were randomly selected from the screened males and divided into three groups and fed the different test foods (male Tamogitake group, male Moringa group, and male Rice bran group) (Figure 1). Moreover, 87 females were randomly selected from the screened females and were divided into three groups (female Tamogitake group, female Moringa group, and female Rice bran group). Assignment to each group by sex was performed so that the age and MMSE score compositions were similar between groups.

### 2.6 Test foods

Granulated dried Tamogitake fruiting bodies (KOJIRO F001; KJ-MARLIQ Inc., Gunma, Japan), Moringa leaf powder (Bio-I Co., Ltd., Osaka, Japan), and rice bran (Nippon Garlic Corporation, Gunma, Japan) were used.

### 2.7 Food intervention study

After 4 weeks of a pre-observation period, participants in each group consumed the test food for 8 weeks in a specified manner. The daily intake of each test food was determined according to the amount recommended by the distributor. The daily intake of Tamogitake was 3 g, with 1 g taken with water at each meal three times a day. The daily intake of Moringa was 3 g, which was taken once a day before dinner by suspending it in water. The daily intake of rice bran was 15 g, with 5 g taken with water at each meal three times a day.

Participants were instructed to maintain the same living conditions (sleep, diet, and lifestyle) as before the start of the study and avoid consuming health foods (such as drinks containing dietary fiber), supplements (containing lactic acid bacteria, oligosaccharides, etc.), or medicines, including intestinal regulators, that affect intestinal function as much as possible during the study period.

Four timepoints were set for observations: 4 weeks before the intake of the test food (BW4: 28 days before the start of intake), immediately before intake (W0: immediately before the start of intake, baseline), 4 weeks after intake (W4: 28 days after the start of intake), and 8 weeks after intake (W8: 56 days after the start of intake). The Cognitrax and intestinal microbiota tests were also conducted. Health status information was collected from participants’ self-reports using a questionnaire at BW4.

### 2.8 Selection of participants for analysis

Among the participants in the food intervention study, the participants for the analysis were selected based on the following inclusion and exclusion criteria.

Inclusion criteria: participants who completed the food intervention study and were classified as not having high cognitive function (NCI score of 110 or less) on the Cognitrax test at baseline.

Exclusion criteria: (1) participants who dropped out of the intervention study, (2) participants with incomplete observations (Cognitrax test and intestinal microbiota test) in the food intervention study, (3) participants were classified as having high cognitive function (NCI score >110) on the Cognitrax test at baseline.

### 2.9 MCI risk estimation

To calculate the MCI risk scores, we used the MCI risk estimation models for males and females reported by Hatayama et al. (7).

### 2.10 Statistical analysis

The paired *t*-test (with Benjamini–Hochberg correction) and Friedman test were performed using R software v.4.1.0 (R Foundation for Statistical Computing, Vienna, Austria) (23). Fisher’s exact test was performed using Excel Statistics (Bell Curve for Excel v3.23; Social Survey Research Information Co., Ltd., Tokyo, Japan). Statistical significance was set at *p* < 0.05.

## 3 Results

### 3.1 Selection of participants for analysis

Based on the MMSE results, participants for the food intervention study were screened from the initial group of 1,229 Japanese individuals aged 60–79 years, and those selected were assigned to the Tamogitake, Moringa, and Rice bran groups by sex according to the test food they consumed (Figure 1). Each group underwent a 4-week pre-intervention observation before starting the intervention and then consumed the test food for 8 weeks. The aim of this pre-intervention observation period was to suppress the impact of increased scores on the Cognitrax test due to familiarity with the test and of unconscious behavioral changes due to study participation on the study results.

Participants who had a Cognitrax NCI score >110 at baseline (W0) and were classified as having high cognitive function were excluded from the analysis due to the difficulty of observing further improvements in cognitive function (the NCI score is the average of the five domain scores for composite memory, psychomotor speed, reaction time, complex attention, and cognitive flexibility, and is an indicator of overall cognitive function) (number excluded: male Tamogitake group, *n* = 8; male Moringa group, *n* = 8; male Rice bran group, *n* = 12; female Tamogitake group, *n* = 3; female Moringa group, *n* = 7; female Rice bran group, *n* = 1). In addition, participants who dropped out of the intervention study due to personal reasons (male Tamogitake group, *n* = 2; male Moringa group, *n* = 3; male Rice bran group, *n* = 5; female Tamogitake group, *n* = 3; female Moringa group, *n* = 2; female Rice bran group, *n* = 2), and participants with incomplete Cognitrax testing (male Tamogitake group, *n* = 1; male Rice bran group, *n* = 1) and with incomplete stool sample collection (male Tamogitake group, *n* = 1; male Moringa group, *n* = 1; male Rice bran group, *n* = 1) were also excluded from the analysis. The number of participants analyzed in each group is shown in Figure 1. During the intervention period, the daily intake rate of the test food was ≥91.1%, except for one participant (71.4%) in the male Rice bran group. No adverse effects were reported by participants in this food intervention study, including those who dropped out of the study.

### 3.2 Responders and non-responders in cognitive function improvement

**FIGURE 2:**
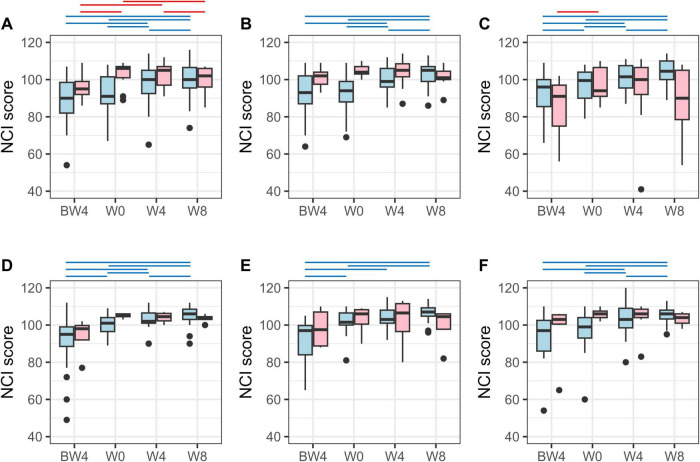
Neurocognitive index (NCI) score on Cognitrax test. NCI score variation for responders (light blue) and non-responders (pink) in **(A)** the male Tamogitake, **(B)** male Moringa, **(C)** male Rice bran, **(D)** female Tamogitake, **(E)** female Moringa, and **(F)** female Rice bran groups. BW4, 4 weeks before intervention; W0, just before intervention; W4, 4 weeks after intervention; W8, 8 weeks after intervention. Blue (responder) and red (non-responder) horizontal lines indicate *p* < 0.05 (after correction by the Benjamini–Hochberg method, paired *t*-test).

Among the analyzed participants in each group, some showed an improvement in their NCI score on the Cognitrax test after the 8-week intervention, while others did not (Figure 2). Therefore, we defined participants whose NCI score at W8 was higher than that at W0 (baseline) as responders; these showed improvement in cognitive function as a result of the 8-week intervention with the test food. For the NCI scores of the responders (excluding the female Moringa), a significant increase in scores was observed at W4 compared to W0, and the significant difference was maintained at W8 (Figure 2). For female Moringa responders, a significant increase in NCI scores was observed at W8, requiring more time than the other groups. In contrast, those with the same or lower NCI scores at W8 compared to W0 were defined as non-responders; these did not show any improvement in cognitive function as a result of the test food intervention.

Table 1 shows the results of the changes in Cognitrax test scores with the test food intervention for each responder (Res) and non-responder (Non) subgroup (details of the Cognitrax test results for each subgroup are shown in Supplementary Tables 1–6). Among the Res subgroups, there were differences in the cognitive domains (composite memory, verbal memory, psychomotor speed, reaction time, complex attention, cognitive flexibility, and executive function) that were improved and the timing of those observations, depending on sex and the test foods. In contrast, the only cognitive domain in which Non subgroups showed a significant improvement in scores was executive function at W8 in the female Tamogitake group. Each Non subgroup showed little improvement in cognitive function, despite the intake of the test food. Visual memory, simple attention, and motor speed did not improve significantly in any of the subgroups.

**TABLE 1:** Score variation in each cognitive domain by Cognitrax test due to the intervention of test foods.

Cognitive domain	Time	Tamogitake group				Moringa group				Rice bran group			
		Male		Female		Male		Female		Male		Female	
		Res	Non	Res	Non	Res	Non	Res	Non	Res	Non	Res	Non
		(23)	(13)	(19)	(4)	(25)	(11)	(16)	(4)	(18)	(11)	(19)	(7)
Neurocognitive index (NCI)	W4	+	−	+	−	+	−	−	−	+	−	+	−
	W8	+	−	+	−	+	−	+	−	+	−	+	−
Composite memory	W4	−	−	−	−	−	−	−	−	−	−	+	−
	W8	−	−	−	−	−	−	+	−	+	−	+	−
Verbal memory	W4	−	−	−	−	−	−	−	−	−	−	+	−
	W8	−	−	−	−	+	−	+	−	+	−	+	−
Visual memory	W4	−	−	−	−	−	−	−	−	−	−	−	−
	W8	−	−	−	−	−	−	−	−	−	−	−	−
Psychomotor speed	W4	−	−	−	−	−	−	−	−	−	−	+	−
	W8	+	−	−	−	+	−	−	−	−	−	−	−
Reaction time	W4	−	−	−	−	−	−	−	−	−	−	−	−
	W8	−	−	−	−	+	−	−	−	−	−	−	−
Complex attention	W4	+	−	−	−	+	−	−	−	−	−	−	−
	W8	+	−	−	−	+	−	−	−	+	−	+	−
Cognitive flexibility	W4	+	−	+	−	+	−	−	−	+	−	+	−
	W8	+	−	+	−	+	−	+	−	+	−	+	−
Processing speed	W4	+	−	−	−	−	−	−	−	−	−	+	−
	W8	+	−	+	−	+	−	−	−	+	−	−	−
Executive function	W4	−	−	+	−	+	−	−	−	+	−	+	−
	W8	+	−	+	+	+	−	+	−	+	−	+	−
Simple attention	W4	−	−	−	−	−	−	−	−	−	−	−	−
	W8	−	−	−	−	−	−	−	−	−	−	−	−
Motor speed	W4	−	−	−	−	−	−	−	−	−	−	−	−
	W8	−	−	−	−	−	−	−	−	−	−	−	−

Res and Non denote responder and non-responder subgroups, respectively. Numbers in parentheses show the number of participants in the subgroup.+: significantly improved after 4 weeks (W4) or 8 weeks (W8) of intervention compared to just before intervention (W0). −: did not significantly improve at W4 or W8 compared to W0. The paired *t*-test was performed (statistical significance: *p* < 0.05, after correction by the Benjamini–Hochberg method).

### 3.3 Intestinal bacteria whose relative abundance varied with test food intervention

For each Res and Non subgroup, we analyzed the intestinal bacteria whose relative abundances varied according to the test food intervention. A Friedman test was performed on the CLR-transformed abundances at W0, W4, and W8 for each of the intestinal bacterial taxa, and taxa with significant differences were defined as those that varied after the test food intervention. However, minor taxa with a detection rate of less than 25% within the subgroups analyzed at W0 were excluded from the analysis. The taxa of intestinal bacteria whose relative abundances varied with the test food intervention for each subgroup are shown in Supplementary Tables 7–18. A comparison of the variable intestinal bacterial taxa between the Res and Non subgroups of the same sex group showed that most of them were different (Table 2). The only common bacterium, including its variability trend, between the Res and Non subgroups of the same sex group was *Parabacteroides* in the male Rice bran group.

**TABLE 2:** Taxa of intestinal bacteria whose relative abundance varied with test food intervention.

Taxa (genus level)	Tamogitake group				Moringa group				Rice bran group			
	**Male**		**Female**		**Male**		**Female**		**Male**		**Female**	
	**Res**	**Non**	**Res**	**Non**	**Res**	**Non**	**Res**	**Non**	**Res**	**Non**	**Res**	**Non**
*Adlercreutzia*	−	−	−	−	−	−	4	−	3	−	−	1
*Agathobaculum*	1	−	1	−	1	−	1	−	−	−	−	−
*Anaerobutyricum*	2	−	−	−	2	−	−	−	2	−	−	−
*Anaerostipes*	−	−	−	−	2	−	−	−	−	2	−	−
*Anaerotignum*	−	−	−	−	−	−	−	2	−	2	−	−
*Bacteroides*	−	−	−	−	2	−	−	−	−	−	−	4
*Blautia*	2	−	2	−	−	−	−	−	2	−	−	−
*Collinsella*	1	−	1	−	−	−	1	−	−	1	−	−
*Coprococcus*	−	−	−	−	−	2	−	2	−	−	−	−
*Dysosmobacter*	−	−	−	2	1	−	−	−	−	−	−	−
*Enterocloster*	3	−	1	−	2	−	−	−	−	−	−	−
*Erysipelatoclostridium*	2	−	−	−	−	−	−	−	−	3	−	−
*Faecalibacterium*	−	−	−	−	−	−	1	−	1	−	1	−
*Fusobacterium*	−	−	−	1	−	−	−	1	−	−	−	−
*Intestinimonas*	−	−	−	−	1	−	1	−	−	−	−	1
*Mediterraneibacter*	−	−	−	−	−	2	−	2	−	−	−	4
*Megasphaera*	−	−	−	1	−	2	−	−	−	−	−	−
*Parabacteroides*	2	−	2	−	−	−	−	−	2	2	2	−
*Phascolarctobacterium*	2	−	−	3	2	−	−	−	−	−	−	−
*Ruminococcus*2	4	2	−	−	−	−	−	−	2	−	−	−
*Sellimonas*	−	−	−	2	−	−	−	−	−	1	−	−
Unclassified	−	1	−	−	1	−	−	−	1	−	1	−

Variable intestinal bacterial taxa in several of the responder (Res) and non-responder (Non) subgroups are shown. Each taxon showed a detection rate of at least 25% in the subgroup immediately before ingestion (W0). Numbers 1–4 indicate a variable trend in relative abundance. 1: increased from W0 to W4, decreased from W4 to W8; 2: decreased from W0 to W4, increased from W4 to W8; 3: increased from W0 to W4, increased from W4 to W8; 4: decreased from W0 to W4, decreased from W4 to W8. −: relative abundance did not vary.

Between the male and female Res subgroups fed identical test foods, some similarities were found in the taxa of intestinal bacteria whose relative abundances varied (Table 2). *Parabacteroides*, *Agathobaculum*, *Collinsella*, and *Blautia* were common among male and female Tamogitake Res subgroups, including trends in variability. *Agathobaculum* and *Intestinimonas* were common among male and female Moringa Res subgroups, and *Parabacteroides*, Unclassified (a taxon comprising ASVs that could not be classified), and *Faecalibacterium* were common among male and female Rice bran Res subgroups, including their variability trends. In contrast, among male and female Non subgroups fed the same test food, only the Moringa Non subgroup showed commonality in some of the intestinal bacterial taxa. The common taxa were *Mediterraneibacter* and *Coprococcus*.

In males, *Anaerobutyricum* was common to all Res subgroups as a taxon of intestinal bacteria whose relative abundance varied (Table 2). *Agathobaculum* and *Phascolarctobacterium* were common among the male Tamogitake and Moringa Res subgroups, *Parabacteroides* and *Blautia* were common among the male Tamogitake and Rice bran Res subgroups, and Unclassified was common among the male Moringa and Rice bran Res subgroups, including their variability trends. In contrast, when the male Non subgroups were compared with each other, there was no commonality in the taxa of intestinal bacteria whose relative abundances varied, and they all differed.

In females, *Agathobaculum* and *Collinsella* were common among the Res subgroups of Tamogitake and Moringa, *Parabacteroides* among the Res subgroups of Tamogitake and Rice bran, and *Faecalibacterium* among the Res subgroups of Moringa and Rice bran as the taxa of intestinal bacteria whose relative abundances varied. In contrast, in the female Non subgroup, *Fusobacterium* was the only common intestinal bacterial taxon whose relative abundance varied between the Tamogitake and Moringa Non subgroups, including its variability trend.

These results indicated that the improvement of cognitive function is associated with variations in the relative abundances of certain intestinal bacteria resulting from the intake of test foods, some of which are common to different sexes and different test foods taken.

### 3.4 Relationship between intestinal microbiota composition and improved cognitive function

In this food intervention study, despite consuming the same test food, some participants exhibited variations in the relative abundances of certain intestinal bacteria and were thus classified as responders, while others did not and thus were classified as non-responders. The composition of the intestinal microbiota immediately before the intake of the test foods is not irrelevant to the variation in the respective intestinal bacteria after intake. This means that the composition of the intestinal microbiota immediately before intake of the test foods could determine whether cognitive function improved after intake. Therefore, to confirm the relationship between the composition of intestinal microbiota and cognitive function improvement immediately before intake of the test food, the β-diversity of responders and non-responders at W0 was visualized by NMDS.

**FIGURE 3:**
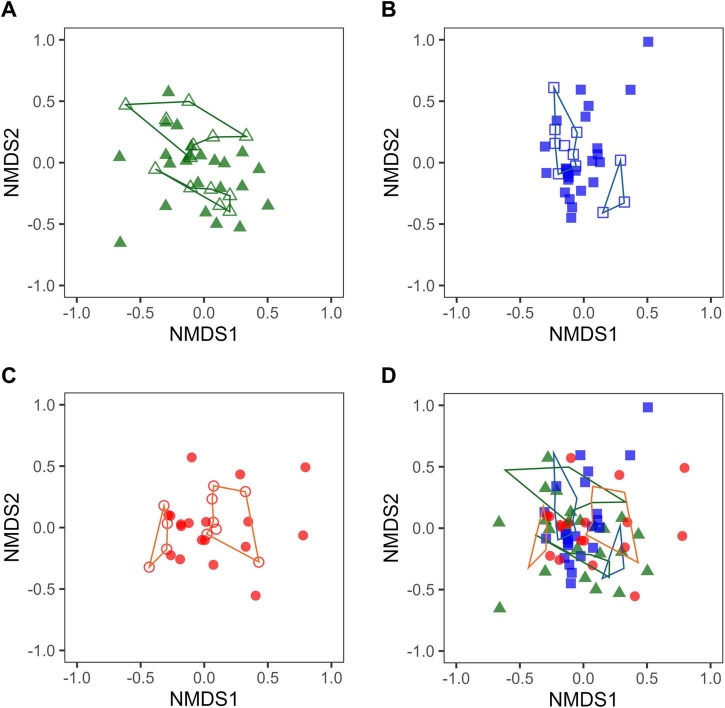
Non-metric multidimensional scaling (NMDS) plots based on Bray–Curtis index of male intestinal microbiota just before intervention (W0). Green, blue, and red show the male Tamogitake [panels **(A,D)**], Moringa [panels **(B,D)**), and Rice bran [panels **(C,D)**] groups, respectively. Closed and open plots show responder and non-responder, respectively. The lines connect non-responder plots. The NMDS plots in panels **(A–D)** are on the same scale (Stress: 0.2377783).

**FIGURE 4:**
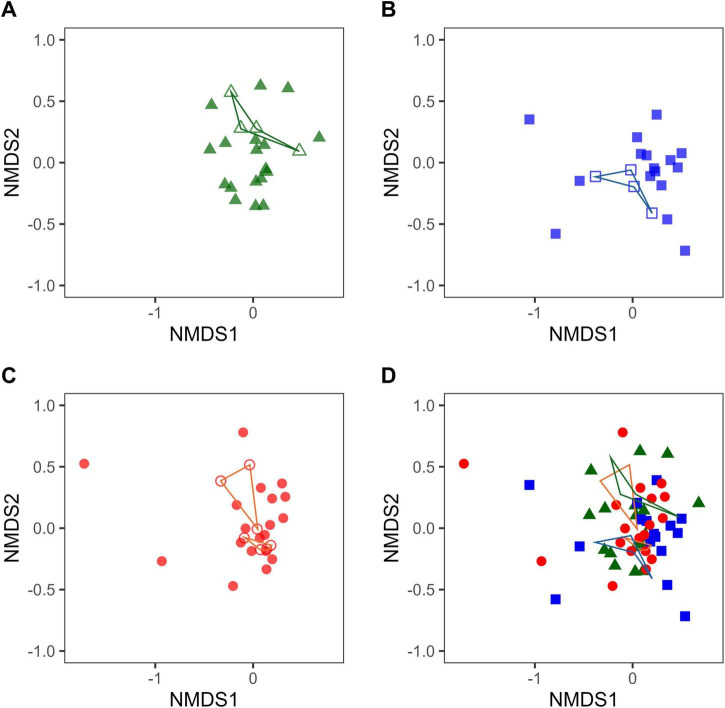
Non-metric multidimensional scaling (NMDS) plots based on Bray–Curtis index of female intestinal microbiota just before intervention (W0). Green, blue, and red show the female Tamogitake [panels **(A,D)**], Moringa [panels **(B,D)**], and Rice bran [panels **(C,D)**] groups, respectively. Closed and open plots show responder and non-responder, respectively. The lines connect non-responder plots. The NMDS plots in panels **(A–D)** are on the same scale (Stress: 0.1999232).

The NMDS plots for each test food intervention group at W0 showed that the non-responder plots were in close proximity and could be captured as one or two small clusters (clusters enclosed by lines in Figures 3A–C, 4A–C). Since the positioning of the NMDS plots reflects similarities in the composition of the intestinal microbiota, the composition of intestinal microbiota in plots belonging to each small cluster was considered to be similar. In other words, one or two similar patterns in the intestinal microbiota composition in non-responders were observed in each test food intervention group. In the NMDS plot, which merged the plots of the three test food intervention groups by sex, a discrepancy was observed in the regions of the non-responder clusters in each group (Figures 3D, 4D). This discrepancy could reflect the existence of differences in the compositional pattern of intestinal microbiota that do not respond to the test foods. These results suggest that if the test food can be selected in advance to suit the composition of the intestinal microbiota of the intake candidate, it is possible to avoid getting a non-response outcome and increase the possibility of obtaining a response outcome.

### 3.5 Relationship between cognitive function improvement and MCI risk by test food intervention

We have reported in previous studies that the characteristic dysbiosis of the intestinal microbiota in MCI may persist for long periods of time (over 10 or 20 years), ultimately leading to cognitive decline (7). Therefore, we determined whether the improvement in cognitive function observed in responders of this food intervention study was associated with an improvement in dysbiosis of the intestinal microbiota, which is characteristic of MCI.

We previously reported risk estimation models for MCI in males and females using intestinal microbiota data (7). These models were constructed using structural equation modeling to reflect the characteristic dysbiosis of the intestinal microbiota in MCI. MCI risk estimation models use intestinal microbiota data as input and output an MCI risk score (a value between 0 and 1). A reduction in the MCI risk score suggests an improvement in dysbiosis of the intestinal microbiota, which is characteristic of MCI. Using these MCI risk estimation models, the MCI risk scores for the participants in this food intervention study were calculated.

Changes in the MCI risk scores in the Res and Non subgroups for each test food from W0 to W8 were evaluated. Consequently, in each Res subgroup, the percentage of those with reduced MCI risk scores at W8 compared to those at W0 ranged from 52.0% to 73.7% (Table 3). However, these percentages were not significantly different from those of the Non subgroups. In addition, correlations were evaluated separately by sex for the variation in NCI and MCI risk scores from W0 to W8, and no significant correlations were observed (male: correlation coefficient 0.017, *p*-value 0.869; female: correlation coefficient 0.041, *p*-value 0.737). These results suggested that the improvement in the cognitive function of responders is not directly related to the improvement in dysbiosis of the intestinal microbiota.

**TABLE 3:** Percentage of participants with reduced MCI risk score at W8 compared to that at W0 for each test food among responders (Res) and non-responders (Non).

Group		Percentage of participants with reduced MCI risk score		*p*-Value
		**Res subgroup**	**Non subgroup**	
Male	Tamogitake	60.9	38.5	0.299
	Moringa	52.0	72.7	0.295
	Rice bran	61.1	72.7	0.694
Female	Tamogitake	57.9	75.0	1.000
	Moringa	56.3	50.0	1.000
	Rice bran	73.7	71.4	1.000

The *p*-values were obtained by Fisher’s exact test.

## 4 Discussion

This food intervention study was conducted to verify whether a food intervention approach targeting the intestinal microbiota could improve the cognitive function of elderly Japanese individuals with suspected MCI, with the goal of preventing dementia and MCI. The International Conference on Harmonization of Technical Requirements for Registration of Pharmaceuticals for Human Use E10 guidelines state that dementia is difficult to detect intervention effects in a placebo-controlled trial (24). In addition, no single dietary intervention has been clearly proven to effectively prevent cognitive decline or dementia in randomized controlled trials in the past (11). Furthermore, there was a concern that the intake of the placebo itself would affect the intestinal microbiota. Therefore, we did not include placebo-controlled studies.

Herein, the participants were eventually divided into responder and non-responder subgroups, with the former showing cognitive function improvement and the latter not showing improvement. The effects of the test food on cognitive function improvement via the intestinal microbiota could be analyzed by comparing these subgroups. To better understand the effect of food on the intestinal microbiota, food intervention studies comparing responders (those who show the expected effect) and non-responders (those who do not show the expected effect) should be conducted in the future.

This food intervention study was designed to conduct a second Cognitrax test at baseline (W0). When cognitive function tests are repeated, it is possible that the score will improve in the second test compared to that in the first test due to practice effects (familiarity with the test) (25, 26). A pre-observation period was thus established to reduce the impact of this practice effect. In fact, each score on the Cognitrax test tended to increase at W0 compared to that at BW4, suggesting the importance of setting the pre-observation period. Although the impact of practice effects on the third and fourth tests could not be completely eliminated, the fact that non-responders were observed suggests that the impact was not as great as that between the first and second tests, confirming the reliability of the test results.

Based on the MMSE results, participants without high cognitive function were screened from among the recruited participants and assigned to each food intervention group. However, some participants were excluded from the analysis because they were classified as having high cognitive function according to the Cognitrax test at W0 (Figure 1). This may have been due to the improvement in scores caused by the practice effects in the second test. In addition, participants who were excluded from the analysis may have been classified as having high cognitive function by the Cognitrax test, which was corrected for age. The Cognitrax test scores were corrected for age in 5-year increments, but the MMSE score was not.

In the food intervention study, unlike non-responders, responders showed variations in the relative abundances of intestinal bacteria belonging to specific taxa due to food intake, some of which were common across sexes and the different test foods consumed. These results indicate that cognitive function may be improved by variations in specific intestinal bacteria due to food intake. However, improving cognitive function via changes in specific intestinal bacteria is likely to depend on the compatibility of the food with the intestinal microbiota composition prior to intake.

Many of the intestinal bacteria associated with improved cognitive function tended to show opposite changes in the 4 weeks before and after W4; for example, they increased during the W0–W4 period and then decreased during the W4–W8 period. The resilience of the intestinal microbiota may have affected this. In a study that employed long-term dietary intervention, the relative abundance of intestinal bacteria, which changed once, tended to return to the baseline, as in this study (27). Although the continuous intake of a particular food may cause temporary changes in intestinal bacteria, these bacteria may gradually return to their pre-intake state over time owing to the resilience of the intestinal microbiota.

Non-responders with no improvement in cognitive function were found in each of the groups taking Tamogitake, Moringa, and rice bran. Furthermore, in responders whose cognitive function improved, variations in the relative abundances of specific intestinal bacteria were observed; however, no such variations were observed in non-responders. These findings suggest that it is unlikely that food components directly affect cognitive function and that variations in specific intestinal bacteria are necessary for this improvement. In addition, since improvement in cognitive function was observed 4 weeks after the food intervention (Table 1), it was thought that the trend of changes in intestinal bacteria during the W0–W4 period was strongly associated with this improvement.

Several of the intestinal bacteria that varied in each Res subgroup are reportedly associated with cognitive function. For example, administration of *Agathobaculum* to aged mice induces an increase in mature spines in the cortical pyramidal dendrites, decreases astrocyte reactivity in the cerebral cortex, and an increase in CaMKIIα in the cerebral cortex, thereby improving aging-associated cognitive impairment (28). An increase in mature dendritic spines contributes to stabilize synapses and store memories (28–30). Proinflammatory cytokines released by reactive astrocytes may be harmful to neuronal function and synaptic plasticity in the brain (31–33), and their suppression or inhibition may contribute to neuroprotective effects and improved cognitive function (28, 34, 35). Activation of pCaMKIIα, a key protein kinase important in learning, memory, and neural plasticity, in the cerebral cortex potentially affects memory-related processes (28, 36, 37). *Faecalibacterium* has been reported to be positively correlated with cognitive scores and was decreased in the MCI group compared to that in the healthy group (38). In this previous study, it was also shown that cognitive impairment can be improved by administering a strain of *Faecalibacterium* to an Alzheimer’s disease mouse model. In a pasteurized strain of *Faecalibacterium*, the effects of its administration are potentially related to oxidative stress and mitochondrial function in the brain (38). Further, a study using rats reported that an increase in the relative abundance of *Parabacteroides* negatively correlated with hippocampal-dependent memory function (39). *Parabacteroides* microbial enrichment may alter gene expression in pathways associated with metabolic function, neurodegenerative disease, and dopaminergic signaling (39). Several studies have reported that a high relative abundance of *Blautia* is associated with low cognitive performance (40–42). *Phascolarctobacterium* has been reported to increase significantly in MCI (43). However, the biological mechanisms by which *Blautia* and *Phascolarctobacterium* affect cognitive function are unknown. Interestingly, in the Res subgroups of this study, *Agathobaculum* and *Faecalibacterium*, which reportedly have positive effects on cognitive function, tended to increase between W0 and W4, while *Parabacteroides*, *Blautia*, and *Phascolarctobacterium*, which reportedly have negative effects, tended to decrease (Table 2). Therefore, the findings of previous studies support our hypothesis that the improvement in cognitive function in this food intervention study occurred via variations in the intestinal bacteria. However, among the intestinal bacteria whose relative abundances varied in the Res subgroups, there were many taxa whose associations with cognitive function are unknown. For example, *Anaerobutyricum* was a common intestinal bacterium that varied in the male Res subgroups of the three test foods; however, we were unable to find any previous reports on its association with cognitive function. These may have an impact on cognitive function, although it is not yet known.

In our intervention study, changes in the relative abundance of these intestinal bacteria associated with cognitive improvement tended to be transient with a peak at W4. In contrast, improvements in cognitive function tended to be maintained between W4 and W8. These findings suggest that the improvement effect of cognitive function on the host will continue for some time after the peak of these intestinal bacterial changes has passed. However, its duration is unknown, and future long-term intervention studies are needed to know this.

Improvements in cognitive function may also be associated with the degradation of the test food by intestinal bacteria. In a mouse oral administration study, a peptide derived from rice bran improved cognitive function (44). This peptide was prepared by digesting rice bran with thermolysin, a bacteria-derived enzyme. In the rice bran intervention groups, peptides that improved cognitive function may have been produced in the intestines by intestinal bacterial enzymes and may have functioned.

Differences were observed in improved cognitive domains among the Res subgroups and in the taxa of intestinal bacteria that varied in relative abundance. This suggests that the biological mechanisms that contribute to improvement in each cognitive domain may differ, as may the taxa of the intestinal bacteria involved. In contrast, visual memory, simple attention, and motor speed did not show significant improvements in any of the subgroups. These may be cognitive domains that are difficult to improve through mechanisms involving intestinal bacteria.

Between male and female Res subgroups that consumed the same test food, the intestinal bacteria whose relative abundances varied were partially different. This was likely affected by the fact that there are sex differences in the Japanese intestinal microbiota (45). Even among the same-sex Res subgroups, the intestinal bacteria whose relative abundances changed were partially different. Considering that substrate utilization differs depending on the intestinal bacteria, it is natural that intestinal bacteria of different taxa would vary depending on test foods with different components.

Intestinal bacteria affect each other and have a complex symbiotic relationship. While there are intestinal bacteria that directly utilize food components for growth, there are also others whose abundance changes in response to the impact of other bacteria. Because of this complexity, it is difficult to understand the detailed mechanisms underlying intestinal bacterial changes. However, the presence of intestinal bacteria related to improved cognitive function in the intestinal microbiota before food intake is a necessary predisposition to becoming a responder. The NMDS plots immediately before the intervention indicated that there may be one or two similar patterns of non-responder intestinal microbiota composition in each test food intervention group (Figures 3, 4). The intestinal microbiota of non-responders probably lacks the intestinal bacteria associated with improvement in cognitive function, including those that are indirect.

The NMDS plot of W0, which integrated the plots of the three groups by sex (Figures 3D, 4D), indicated that there were differences in the composition patterns of the intestinal microbiota, leading to non-responders depending on the test foods. This suggests that a person who is a non-responder to a particular test food may be a responder to another test food. In other words, one can increase the possibility of improving one’s cognitive function by examining the composition of the intestinal microbiota in advance and then consuming suitable foods. For example, food selection can be performed by adding the desired person’s plot to the NMDS plot of W0, integrating the plots of the three test food groups, and examining the positional relationships between them.

Many of the intestinal bacterial taxa that varied in the Res subgroup of the food intervention study differed from previously reported taxa associated with characteristic dysbiosis of the intestinal microbiota in MCI (7). In addition, the lack of correlation between the variation values of the NCI score and those of the MCI risk score from W0 to W8 suggests that the mechanism of cognitive function improvement via the variation in the intestinal microbiota in this study is not directly related to the mechanism of MCI development via dysbiosis of the intestinal microbiota. When designing the food intervention study, we expected that the intake of the test foods would improve the MCI characteristic of intestinal microbiota dysbiosis and thereby improve cognitive function. However, the results of the food intervention study indicated otherwise. This result was not surprising, given the difference in time scales between intestinal microbiota-associated cognitive decline and improvement. Clearly, the timescale is different, as intestinal microbiota dysbiosis in MCI is expected to cause a gradual decline in cognitive function over a longer period of time, approximately 10–20 years, whereas improvement in cognitive function was observed in a shorter period of time, from 4 to 8 weeks.

Based on the MCI risk score, participants in the food intervention study did not necessarily have the characteristic intestinal microbiota dysbiosis in the MCI group (Supplementary Figure 1). The participants could have included those who did not have high cognitive function owing to certain factors, including natural cognitive decline with aging. The fact that these participants showed improvement in cognitive function also supports the hypothesis that the improvement in cognitive function via variations in specific intestinal microbiota caused by food intake is mechanistically different from the intestinal microbiota dysbiosis hypothesis of MCI.

The different mechanisms of intestinal bacteria-mediated cognitive decline and improvement suggest that any improvement in cognitive function may be temporary and that the risk of cognitive decline may continue. In the long term, improving or preventing the dysbiosis of intestinal microbiota associated with MCI is also important to prevent cognitive function from declining again after improvement. Fortunately, more than half of the responders whose cognitive function improved after consuming the test foods also had reduced MCI risk scores (Table 3). A reduction in MCI risk score could be predicted from the composition of the intestinal microbiota prior to food intake (Supplementary Figures 2, 3). Selecting and taking test foods that can improve cognitive function and reduce the MCI risk score (MCI dysbiosis) based on prior intestinal microbiota test results are more reasonable for those who wish to improve their cognitive function. For example, appropriate food selection can be achieved by adding the desired person’s plot to the NMDS plot of W0 (Supplementary Figure 4), which links the responders of the three test food intervention groups to information on MCI risk reduction and their positional relationships.

Cognitive function improvement has been reported for L-ergothioneine present in Tamogitake (12, 13) and Moringa leaf extracts (14, 15). However, since there were participants who did not show improvement in their cognitive function (non-responders) in this food intervention study, the components reported to improve cognitive function may not have been sufficient at the food level to exert their effects. Alternatively, these components may need to be mediated by intestinal bacteria to be effective. Further studies are required to elucidate the relationship between food components and intestinal bacteria.

Foods that improve cognitive function via changes in intestinal microbiota may exist in addition to the three foods used in this study. Further exploration of these foods is expected to provide more options for improving cognitive function.

The results of this study may be limited to the Japanese population, given that the composition of the intestinal microbiota is affected by ethnicity and geographic location (46). In addition, the participants in the food intervention study were males and females aged 60–79 years with low levels of cognitive function and suspected MCI, and it is not clear whether cognitive improvement occurs in patients with MCI or dementia. To clarify this, future food intervention studies with patients with MCI and dementia are warranted.

This study did not restrict participants’ health status. There were three reasons for this: (1) it was difficult to recruit only disease-free older adults; (2) the cognitively impaired older adults to whom the results of this study might be applied may have some concurrent disease; and (3) this study did not aim to identify intestinal microbiota associated with specific diseases. Therefore, participants in the analysis included those affected by various diseases (Supplementary Table 19). In addition, limitations of this study include the lack of blinding and placebo control groups, which prevent discussion of their effects. In future food intervention trials, it may be necessary to consider controlling participants’ health status, blinding, and placebo control groups, depending on the objectives. Finally, in this food intervention trial, a pre-observation period was included to minimize the influence of practice effect on the Cognitrax test, but it should be noted that this influence may still have been present.

## 5 Conclusion

Intake of either Tamogitake, Moringa, or rice bran may improve cognitive function in elderly Japanese individuals with suspected MCI via variations in specific intestinal bacteria. The intake of these foods may reduce the risk of MCI. However, the occurrence of cognitive improvement and MCI risk reduction may depend on the composition of the intestinal microbiota prior to food intake.

The results of this study may lead to innovative solutions for maintaining and improving cognitive function in the elderly population. This solution is a food intervention approach that targets the intestinal microbiota and differs from conventional pharmaceuticals. Through intestinal microbiota testing, elderly individuals can receive individualized recommendations for appropriate foods to maintain and improve cognitive function, and can work on their own to improve cognitive function and prevent MCI by consuming those foods on an ongoing basis. If social implementation of this solution advances, it is expected to help solve critical issues in Japan’s aging society.

## Data Availability

The datasets presented in this study are not publicly available because of privacy concerns. Requests to access the data should be directed to the corresponding author.

## Footnotes

1
https://zenodo.org/record/4310151#.ZDUBAXbP2Ht
