# Supplementary material for: Effect of a specific food intervention with Tamogitake mushroom, Moringa leaves, or rice bran on intestinal microbiota and cognitive function in elderly Japanese
Source: Front Nutr. 2025 Jul 21;12:1585111. doi: 10.3389/fnut.2025.1585111 (PMC12320749; doi:10.3389/fnut.2025.1585111)
Supplement: Supplementary file 1 [file Data_Sheet_1.pdf]

Supplementary Material

1 Supplementary Figures and Tables

1.1 Supplementary Figures

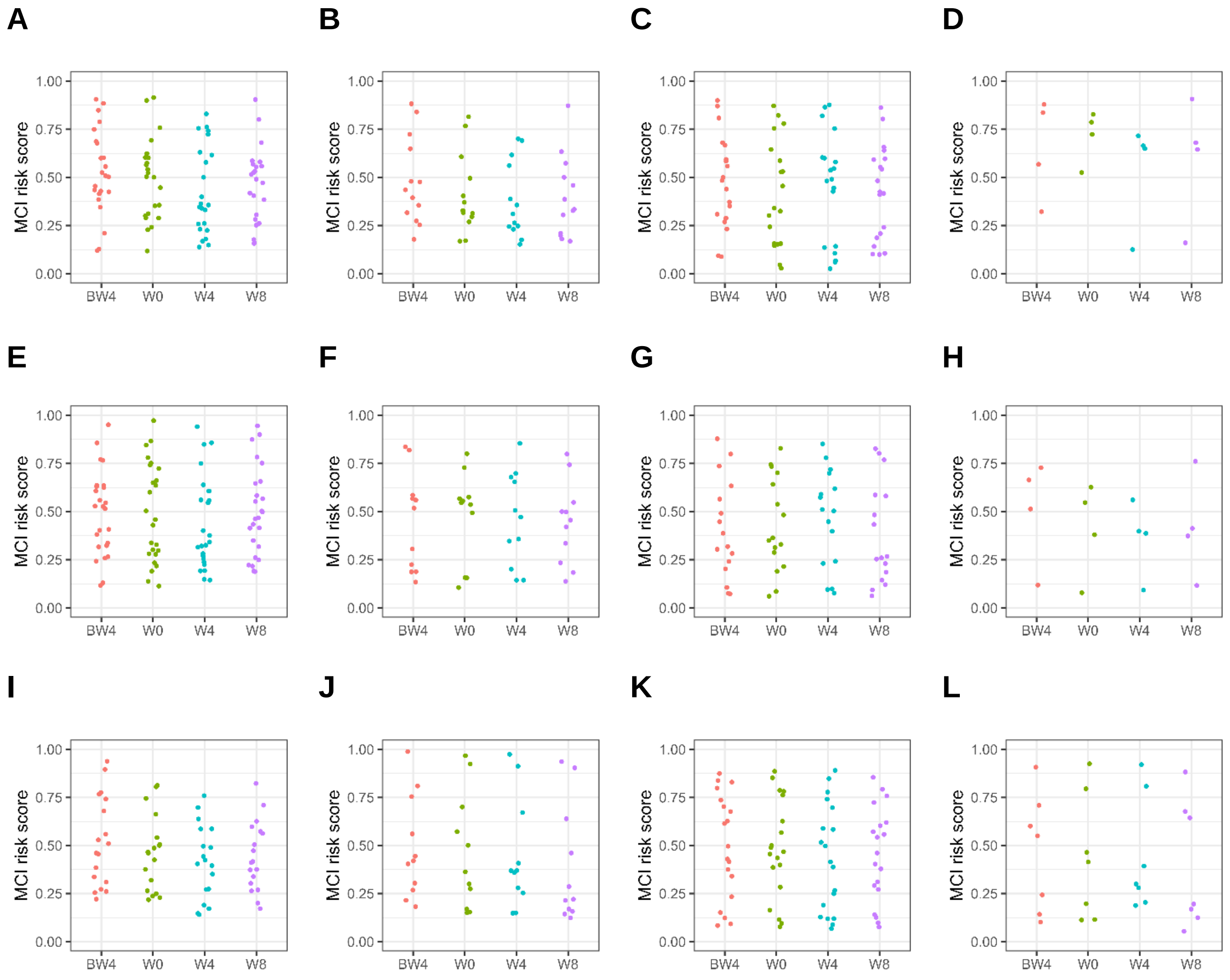

Supplementary Figure S1. Jitter plot of MCI risk scores for participants analyzed in the food intervention trial. (A) male responders of the Tamogitake group; (B) male non-responders of the Tamogitake group; (C) female responders of the Tamogitake group; (D) female non-responders of the Tamogitake group; (E) male responders of the Moringa group; (F) male non-responders of the Moringa group; (G) female responders of the Moringa group; (H) female non-responders of the Moringa group; (I) male responders of the Rice bran group; (J) male non-responders of the Rice bran group; (K) female responders of the Rice bran group; (L) female non-responders of the Rice bran group. BW4: four weeks before food intervention, W0: just before intervention, W4: four weeks after intervention, W8: eight weeks after intervention.

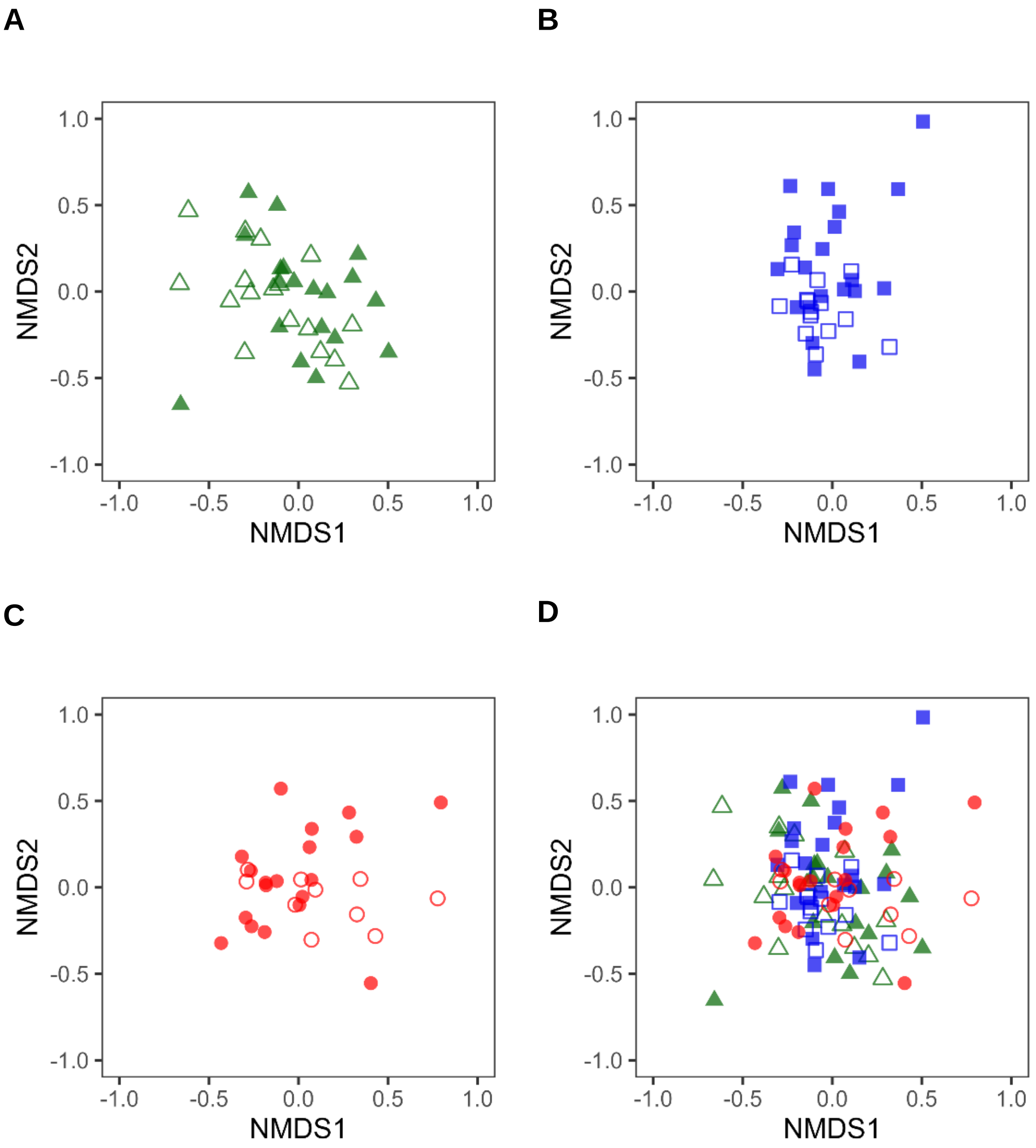

Supplementary Figure S2. Non-metric multidimensional scaling (NMDS) plots based on Bray-Curtis index of male intestinal microbiota just before intervention (W0). Green, blue, and red show the male Tamogitake (panels (A) and (D)), Moringa (panels (B) and (D)), and Rice bran (panels (C) and (D)) groups, respectively. Closed and open plots show participants whose MCI risk scores decreased and did not decrease, respectively.The NMDS plots in (A)-(D) are on the same scale (Stress: 0.2377783).

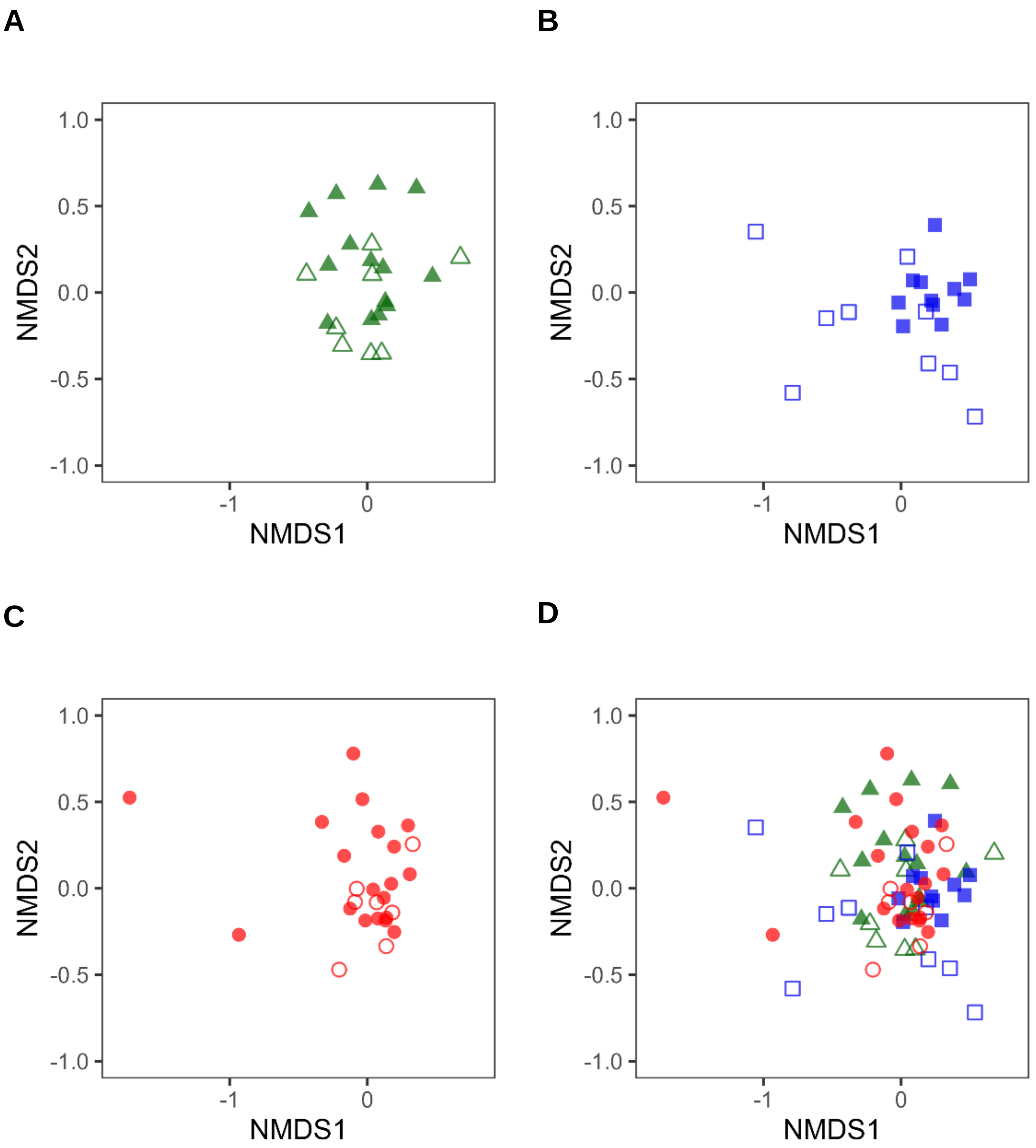

Supplementary Figure S3. Non-metric multidimensional scaling (NMDS) plots based on Bray-Curtis index of female intestinal microbiota just before intervention (W0). Green, blue, and red show the female Tamogitake (panels (A) and (D)), Moringa (panels (B) and (D)), and Rice bran (panels (C) and (D)) groups, respectively. Closed and open plots show participants whose MCI risk scores decreased and did not decrease, respectively. The NMDS plots in (A)-(D) are on the same scale (Stress: 0.1999232).

A B

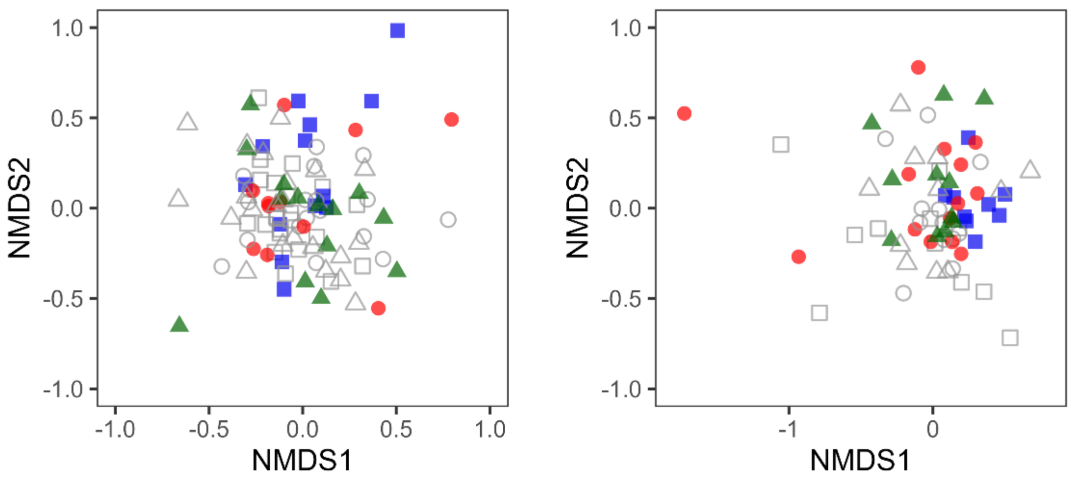

Supplementary Figure S4. Non-metric multidimensional scaling (NMDS) plots based on Bray-Curtis index of male (A) and female (B) intestinal microbiota just before intervention (W0). Closed green triangles, blue squares, and red circles indicate responders with reduced MCI risk in the food intervention test groups for Tamogitake, Moringa, and Rice Bran, respectively. Gray open triangles, squares, and circles indicate responders with no reduction in MCI risk and the non-responders in the food intervention groups for Tamogitake, Moringa, and Rice Bran, respectively. Stress values: (A) 0.2377783; (B) 0.1999232.

1.2 Supplementary Tables

Supplementary Table S1. Cognitive domain scores of the Cognitrax test in the male Tamogitake group.

Cognitive domain	subgroup	Score (mean ± SD)				Δ value			
		BW4	W0	W4	W8	W4		W8	
Neurocognitive index (NCI)	Res	88.5 ± 12.9	92.0 ± 11.7	96.6 ± 12.9*	99.5 ± 9.9*	4.6 ± 7.1	†	7.6 ± 6.7	†
	Non	95.6 ± 6.5	102.8 ± 6.0	102.5 ± 7.2	99.5 ± 7.0*	-0.3 ± 5.6		-3.3 ± 4.5	
Composite memory	Res	68.5 ± 23.8	80.8 ± 13.4	85.0 ± 19.6	89.7 ± 19.3	4.2 ± 15.4		8.8 ± 18.4	†
	Non	85.0 ± 21.2	95.4 ± 13.4	91.5 ± 18.7	92.6 ± 15.3	-3.8 ± 11.0		-2.8 ± 11.8	
Verbal memory	Res	64.4 ± 22.5	84.1 ± 15.6	91.6 ± 19.8	91.6 ± 19.4	7.5 ± 18.8		7.5 ± 21.5	
	Non	80.6 ± 21.7	93.3 ± 18.6	96.2 ± 16.4	93.4 ± 16.8	2.8 ± 10.4		0.1 ± 17.4	
Visual memory	Res	83.6 ± 20.2	84.0 ± 12.1	83.7 ± 17.8	91.7 ± 19.7	-0.3 ± 14.1		7.7 ± 19.9	†
	Non	94.9 ± 17.0	99.8 ± 14.5	89.9 ± 18.3	94.9 ± 12.0	-9.8 ± 15.3		-4.8 ± 14.8	
Psychomotor speed	Res	87.9 ± 22.0	94.6 ± 21.0	98.3 ± 19.7	101.2 ± 14.0*	3.8 ± 8.2		6.6 ± 9.8	
	Non	99.2 ± 11.1	105.7 ± 12.9	103.6 ± 11.6	97.4 ± 18.7	-2.1 ± 16.4		-8.3 ± 26.0	
Reaction time	Res	98.6 ± 27.2	94.7 ± 15.9	96.3 ± 10.6	96.7 ± 11.8	1.7 ± 14.7		2.1 ± 18.1	
	Non	101.3 ± 14.2	100.0 ± 6.7	105.2 ± 12.4	99.6 ± 6.5	5.2 ± 10.7		-0.4 ± 7.0	
Complex attention	Res	94.1 ± 17.3	95.8 ± 18.9	103.1 ± 14.8*	106.8 ± 9.7*	7.3 ± 13.4	†	11.0 ± 13.6	†
	Non	97.4 ± 14.8	108.8 ± 12.5	106.5 ± 13.6	105.8 ± 10.9	-2.3 ± 11.6		-3.1 ± 5.8	
Cognitive flexibility	Res	93.3 ± 14.6	93.9 ± 16.9	99.9 ± 13.2*	103.1 ± 10.8*	6.0 ± 10.4		9.2 ± 10.6	†
	Non	95.4 ± 12.0	104.9 ± 11.9	106.0 ± 13.0	102.2 ± 12.0	1.1 ± 6.7		-2.7 ± 3.7	
Processing speed	Res	102.2 ± 12.2	102.0 ± 13.5	107.6 ± 12.4*	109.6 ± 9.9*	5.6 ± 8.4	†	7.6 ± 8.3	†
	Non	108.4 ± 9.6	114.3 ± 8.6	113.4 ± 6.8	114.1 ± 10.6	-0.9 ± 6.5		-0.2 ± 6.5	
Executive function	Res	94.3 ± 15.0	95.8 ± 15.3	100.0 ± 12.8	103.0 ± 10.7*	4.2 ± 10.3		7.2 ± 9.8	†
	Non	97.8 ± 10.5	104.8 ± 11.7	107.3 ± 12.3	102.3 ± 11.7*	2.5 ± 5.0		-2.5 ± 3.4	
Simple attention	Res	81.2 ± 77.0	96.8 ± 23.3	99.3 ± 16.7	98.8 ± 15.0	2.4 ± 25.0		2.0 ± 21.8	
	Non	104.4 ± 11.0	108.7 ± 3.1	104.3 ± 7.3	106.5 ± 6.5	-4.4 ± 7.1		-2.2 ± 6.5	
Motor speed	Res	82.1 ± 28.4	91.0 ± 23.9	92.3 ± 23.1	95.0 ± 15.2	1.3 ± 7.0		4.0 ± 12.4	
	Non	93.2 ± 14.5	98.1 ± 13.9	95.5 ± 14.3	86.6 ± 26.6	-2.6 ± 19.1		-11.5 ± 33.5	

Res and Non denote the responder and non-responder subgroups, respectively. BW4: four weeks before food intervention; W0: just before intervention; W4: four weeks after intervention; W8: eight weeks after intervention. Paired t-tests were performed for within-group comparisons (W0 vs. W4 and W0 vs. W8) (*, indicates *p*-value < 0.05, after correction by the Benjamini-Hochberg method). Δ value represents the change in score from the baseline W0, and an unpaired t-test was performed between the responders and non-responders (†, *p*-value less than 0.05).

Supplementary Table S2. Cognitive domain scores of the Cognitrax test in the female Tamogitake group.

Cognitive domain	subgroup	Score (mean ± SD)				Δ value	
		BW4	W0	W4	W8	W4	W8
Neurocognitive index (NCI)	Res	90.3 ± 15.2	100.2 ± 5.9	102.6 ± 5.6*	104.7 ± 5.4*	2.5 ± 3.6	4.5 ± 3.1 †
	Non	93.8 ± 9.8	105.0 ± 1.2	104.0 ± 2.7	103.5 ± 2.2	-1.0 ± 3.2	-1.5 ± 1.1
Composite memory	Res	86.7 ± 20.4	95.6 ± 16.2	98.2 ± 15.8	98.6 ± 14.7	2.6 ± 11.7 †	3.1 ± 13.4 †
	Non	94.5 ± 12.0	106.5 ± 7.7	101.8 ± 6.6	91.0 ± 12.1	-4.8 ± 2.9	-15.5 ± 5.6
Verbal memory	Res	86.6 ± 25.4	99.4 ± 19.5	102.7 ± 16.9	100.3 ± 17.4	3.3 ± 13.9	0.9 ± 18.6 †
	Non	91.8 ± 14.3	112.3 ± 1.8	103.8 ± 9.8	94.0 ± 11.6	-8.5 ± 8.8	-18.3 ± 10.1
Visual memory	Res	92.1 ± 14.2	93.3 ± 11.4	94.8 ± 19.7	98.2 ± 12.5	1.5 ± 15.9	4.8 ± 10.3
	Non	100.3 ± 7.4	98.5 ± 12.7	99.5 ± 6.2	91.0 ± 13.9	1.0 ± 8.9	-7.5 ± 8.6
Psychomotor speed	Res	94.2 ± 18.7	101.2 ± 9.4	101.4 ± 9.2	103.7 ± 7.5	0.3 ± 6.7	2.6 ± 5.6
	Non	94.5 ± 5.2	100.3 ± 3.3	101.0 ± 8.5	105.8 ± 7.9	0.8 ± 6.4	5.5 ± 5.1
Reaction time	Res	90.8 ± 16.1	98.7 ± 14.7	96.8 ± 10.0	100.1 ± 9.9	-1.8 ± 12.0	1.4 ± 10.5
	Non	96.8 ± 4.1	101.3 ± 8.3	96.0 ± 11.4	101.3 ± 6.9	-5.3 ± 4.9	0 ± 3.7
Complex attention	Res	86.0 ± 45.2	104.7 ± 11.9	110.6 ± 5.8	112.2 ± 4.1	5.9 ± 12.0	7.5 ± 11.1 †
	Non	92.3 ± 20.5	110.5 ± 4.6	112.0 ± 7.5	109.5 ± 3.8	1.5 ± 5.1	-1.0 ± 2.9
Cognitive flexibility	Res	94.1 ± 15.2	100.8 ± 9.9	106.3 ± 5.9*	108.5 ± 4.7*	5.5 ± 8.3	7.6 ± 8.0 †
	Non	89.8 ± 20.1	107.0 ± 1.2	110.3 ± 5.5	110.3 ± 1.3	3.3 ± 4.4	3.3 ± 1.1
Processing speed	Res	102.7 ± 11.5	107.3 ± 9.8	107.8 ± 8.9	112.6 ± 8.5*	0.6 ± 7.6	5.4 ± 7.8
	Non	108.8 ± 6.4	109.3 ± 7.5	109.3 ± 10.8	117.0 ± 4.9	0 ± 10.0	7.8 ± 12.1
Executive function	Res	93.9 ± 15.2	101.5 ± 7.8	105.9 ± 6.1*	108.1 ± 4.5*	4.4 ± 5.8	6.6 ± 5.8
	Non	89.8 ± 20.1	107.0 ± 1.4	110.5 ± 5.5	110.8 ± 1.5*	3.5 ± 4.6	3.8 ± 0.4
Simple attention	Res	25.7 ± 229.7	101.6 ± 9.9	103.1 ± 9.0	103.5 ± 9.2	1.5 ± 11.5	1.9 ± 12.7
	Non	92.8 ± 18.4	100.0 ± 5.7	102.3 ± 11.8	92.5 ± 15.9	2.3 ± 14.0	-7.5 ± 12.4
Motor speed	Res	90.1 ± 24.1	96.5 ± 10.6	96.1 ± 9.4	96.5 ± 8.9	-0.4 ± 5.7	-0.1 ± 6.0
	Non	87.5 ± 10.2	93.3 ± 7.3	93.8 ± 8.1	94.3 ± 7.9	0.5 ± 4.1	1.0 ± 4.3

Res and Non denote the responder and non-responder subgroups, respectively. BW4: four weeks before food intervention; W0: just before intervention; W4: four weeks after intervention; W8: eight weeks after intervention. Paired t-tests were performed for within-group comparisons (W0 vs. W4 and W0 vs. W8) (*, indicates *p*-value < 0.05, after correction by the Benjamini-Hochberg method). Δ value represents the change in score from the baseline W0, and an unpaired t-test was performed between the responders and non-responders (†, *p*-value less than 0.05).

Supplementary Table S3. Cognitive domain scores of the Cognitrax test in the male Moringa group.

Cognitive domain	subgroup	Score (mean ± SD)				Δ value	
		BW4	W0	W4	W8	W4	W8
Neurocognitive index (NCI)	Res	92.3 ± 11.7	93.0 ± 10.7	99.9 ± 7.6*	102.2 ± 7.2*	6.9 ± 7.9 †	9.2 ± 7.2 †
	Non	100.9 ± 4.3	104.8 ± 3.2	103.8 ± 7.3	101.5 ± 5.0	-1.0 ± 6.7	-3.3 ± 3.6
Composite memory	Res	80.6 ± 19.5	86.5 ± 16.5	89.2 ± 17.4	90.4 ± 17.2	2.8 ± 15.3	4.0 ± 13.9 †
	Non	102.6 ± 15.7	106.7 ± 8.7	103.7 ± 11.0	95.5 ± 15.1*	-3.0 ± 11.4	-11.2 ± 10.3
Verbal memory	Res	77.3 ± 20.1	84.4 ± 21.1	91.1 ± 19.4	93.6 ± 19.5*	6.6 ± 22.9	9.2 ± 18.5 †
	Non	98.6 ± 17.0	102.5 ± 12.6	102.6 ± 11.1	98.5 ± 14.1	0.2 ± 16.9	-3.9 ± 10.5
Visual memory	Res	90.7 ± 17.8	93.2 ± 16.0	91.2 ± 13.7	89.9 ± 15.0	-2.0 ± 12.1	-3.4 ± 13.3 †
	Non	107.1 ± 12.6	109.7 ± 8.9	103.8 ± 12.7	94.6 ± 16.9*	-5.9 ± 11.8	-15.1 ± 14.4
Psychomotor speed	Res	98.0 ± 12.5	96.8 ± 16.1	104.0 ± 8.8	105.2 ± 9.6*	7.2 ± 15.6	8.4 ± 16.4 †
	Non	103.8 ± 15.1	108.1 ± 9.3	99.5 ± 24.3	100.2 ± 24.2	-8.5 ± 21.9	-7.9 ± 21.0
Reaction time	Res	94.0 ± 17.9	93.4 ± 12.2	98.0 ± 14.9	102.2 ± 14.4*	4.6 ± 10.6	8.8 ± 14.0
	Non	95.4 ± 10.7	96.1 ± 8.4	99.5 ± 6.0	98.6 ± 8.2	3.5 ± 5.8	2.5 ± 4.7
Complex attention	Res	94.8 ± 21.2	92.6 ± 32.3	105.4 ± 11.8*	108.3 ± 11.0*	12.8 ± 28.3	15.6 ± 29.6 †
	Non	103.4 ± 8.2	109.4 ± 5.3	110.0 ± 5.1	109.3 ± 5.1	0.6 ± 7.0	-0.1 ± 5.9
Cognitive flexibility	Res	94.3 ± 13.1	95.6 ± 11.5	102.5 ± 8.9*	105.2 ± 9.3*	6.9 ± 8.8	9.6 ± 6.7 †
	Non	99.5 ± 5.9	103.3 ± 7.2	106.1 ± 7.4	104.7 ± 6.4	2.8 ± 8.3	1.5 ± 6.1
Processing speed	Res	104.6 ± 11.2	108.6 ± 10.5	109.5 ± 9.2	112.9 ± 10.5*	0.9 ± 7.5	4.3 ± 8.2
	Non	107.7 ± 7.9	111.6 ± 11.1	110.7 ± 8.2	112.9 ± 9.0	-0.9 ± 9.9	1.3 ± 7.6
Executive function	Res	94.8 ± 12.7	95.9 ± 10.4	103.0 ± 8.9*	105.4 ± 9.1*	7.1 ± 8.9	9.5 ± 6.8 †
	Non	100.2 ± 6.7	103.2 ± 7.1	105.6 ± 7.4	104.3 ± 6.3	2.5 ± 8.0	1.1 ± 6.1
Simple attention	Res	76.6 ± 78.9	61.2 ± 142.3	96.8 ± 15.7	102.1 ± 9.3	35.6 ± 139.5	40.9 ± 142.6
	Non	100.0 ± 16.2	106.1 ± 6.4	99.3 ± 10.8	107.9 ± 4.0	-6.8 ± 12.1	1.8 ± 3.9
Motor speed	Res	94.8 ± 13.6	90.7 ± 19.5	98.9 ± 10.6	98.7 ± 9.8	8.2 ± 18.3	8.0 ± 18.9
	Non	99.6 ± 19.7	102.0 ± 10.3	91.1 ± 30.7	90.6 ± 29.8	-10.9 ± 29.7	-11.4 ± 29.0

Res and Non denote the responder and non-responder subgroups, respectively. BW4: four weeks before food intervention; W0: just before intervention; W4: four weeks after intervention; W8: eight weeks after intervention. Paired t-tests were performed for within-group comparisons (W0 vs. W4 and W0 vs. W8) (*, indicates *p*-value < 0.05, after correction by the Benjamini-Hochberg method). Δ value represents the change in score from the baseline W0, and an unpaired t-test was performed between the responders and non-responders (†, *p*-value less than 0.05).

Supplementary Table S4. Cognitive domain scores of the Cognitrax test in the female Moringa group.

Cognitive domain	subgroup	Score (mean ± SD)				Δ value	
		**BW4**	**W0**	**W4**	**W8**	**W4**	**W8**
Neurocognitive index (NCI)	Res	92.9 ± 11.0	101.7 ± 6.9	103.9 ± 5.3	106.4 ± 5.0*	2.3 ± 5.5	4.7 ± 3.3 †
	Non	98.3 ± 9.9	102.8 ± 7.6	101.5 ± 13.1	99.3 ± 10.0	-1.3 ± 5.5	-3.5 ± 2.7
Composite memory	Res	80.2 ± 21.2	90.5 ± 17.5	95.6 ± 16.2	100.5 ± 15.8*	5.1 ± 13.3	10.0 ± 12.5
	Non	102.0 ± 15.8	100.3 ± 9.1	106.3 ± 14.2	103.5 ± 12.3	6.0 ± 5.8	3.3 ± 8.9
Verbal memory	Res	75.3 ± 25.9	88.9 ± 19.5	99.5 ± 19.0	105.7 ± 18.5*	10.6 ± 20.1	16.8 ± 18.5
	Non	93.3 ± 21.4	97.3 ± 10.3	106.3 ± 15.2	106.3 ± 14.2	9.0 ± 6.4	9.0 ± 12.2
Visual memory	Res	91.8 ± 16.0	95.1 ± 16.5	93.6 ± 13.9	95.8 ± 13.6	-1.6 ± 12.8	0.7 ± 14.0
	Non	112.0 ± 7.8	103.8 ± 5.3	105.8 ± 10.3	100.0 ± 7.2	2.0 ± 9.6	-3.8 ± 6.2
Psychomotor speed	Res	101.7 ± 13.9	104.1 ± 15.8	106.8 ± 9.3	107.8 ± 10.9	2.6 ± 9.2	3.7 ± 8.1
	Non	94.5 ± 10.1	101.5 ± 10.0	103.0 ± 9.9	104.3 ± 3.7	1.5 ± 3.4	2.8 ± 10.4
Reaction time	Res	99.3 ± 9.2	99.7 ± 7.4	101.2 ± 8.1	101.1 ± 5.9	1.5 ± 5.6	1.4 ± 6.4
	Non	92.5 ± 7.5	97.0 ± 12.3	97.0 ± 10.6	92.5 ± 5.3	0 ± 3.5	-4.5 ± 8.1
Complex attention	Res	92.4 ± 33.0	109.4 ± 6.7	110.1 ± 8.1	112.6 ± 5.5	0.7 ± 6.2	3.1 ± 4.4
	Non	105.3 ± 10.2	110.3 ± 5.5	103.0 ± 16.8	101.0 ± 17.1	-7.3 ± 12.5	-9.3 ± 13.4
Cognitive flexibility	Res	91.7 ± 19.1	104.8 ± 8.0	106.0 ± 9.9	109.7 ± 6.9*	1.3 ± 6.6	4.9 ± 5.7
	Non	97.8 ± 13.0	105.0 ± 4.8	98.3 ± 16.5	95.0 ± 17.9	-6.8 ± 12.3	-10.0 ± 13.9
Processing speed	Res	102.9 ± 22.8	111.6 ± 9.1	110.9 ± 11.1	113.6 ± 9.7	-0.6 ± 6.5	2.1 ± 5.9
	Non	105.3 ± 8.2	109.0 ± 13.0	117.3 ± 9.3	114.0 ± 12.4	8.3 ± 6.4	5.0 ± 3.3
Executive function	Res	92.3 ± 18.6	104.3 ± 8.3	105.3 ± 10.2	108.8 ± 7.3*	0.9 ± 6.7	4.5 ± 6.0
	Non	98.3 ± 13.2	104.8 ± 5.4	98.0 ± 16.4	94.8 ± 17.8	-6.8 ± 11.8	-10.0 ± 13.6
Simple attention	Res	80.8 ± 88.8	103.4 ± 5.3	102.8 ± 5.7	102.7 ± 9.8	-0.6 ± 6.9	-0.7 ± 10.7
	Non	104.8 ± 9.2	104.8 ± 7.5	107.0 ± 3.7	107.3 ± 4.9	2.3 ± 3.9	2.5 ± 10.2
Motor speed	Res	98.9 ± 14.4	97.7 ± 17.9	101.8 ± 8.7	101.6 ± 12.5	4.1 ± 13.4	3.9 ± 12.2
	Non	88.8 ± 12.6	96.8 ± 6.3	93.5 ± 11.3	96.3 ± 9.3	-3.3 ± 6.6	-0.5 ± 13.0

Res and Non denote the responder and non-responder subgroups, respectively. BW4: four weeks before food intervention; W0: just before intervention; W4: four weeks after intervention; W8: eight weeks after intervention. Paired t-tests were performed for within-group comparisons (W0 vs. W4 and W0 vs. W8) (*, indicates *p*-value < 0.05, after correction by the Benjamini-Hochberg method). Δ value represents the change in score from the baseline W0, and an unpaired t-test was performed between the responders and non-responders (†, *p*-value less than 0.05).

Supplementary Table S5. Cognitive domain scores of the Cognitrax test in the male Rice bran group.

Cognitive domain	subgroup	Score (mean ± SD)				Δ value	
		BW4	W0	W4	W8	W4	W8
Neurocognitive index (NCI)	Res	92.2 ± 11.2	96.9 ± 8.3	100.8 ± 7.5*	103.8 ± 6.8*	3.8 ± 3.3	6.8 ± 3.7 †
	Non	84.9 ± 14.5	97.5 ± 8.7	94.2 ± 18.9	89.2 ± 16.1	-3.3 ± 15.5	-8.3 ± 10.1
Composite memory	Res	83.2 ± 15.4	86.7 ± 14.2	91.4 ± 16.0	95.2 ± 13.5*	4.7 ± 12.6	8.4 ± 11.8
	Non	78.2 ± 19.1	79.5 ± 21.3	87.3 ± 24.4	77.0 ± 27.0	7.8 ± 15.6	-2.5 ± 17.0
Verbal memory	Res	81.6 ± 17.8	86.6 ± 19.2	90.2 ± 24.1	97.3 ± 18.5*	3.7 ± 14.9	10.8 ± 14.9
	Non	78.2 ± 22.4	79.5 ± 21.8	89.3 ± 24.6	78.0 ± 25.8	9.7 ± 16.3	-1.5 ± 15.9
Visual memory	Res	90.8 ± 16.7	91.6 ± 12.4	95.5 ± 11.5	95.8 ± 12.2	3.9 ± 12.1	4.2 ± 13.1
	Non	86.9 ± 15.1	86.5 ± 16.2	89.5 ± 18.8	84.0 ± 21.0	3.0 ± 16.3	-2.5 ± 15.3
Psychomotor speed	Res	94.4 ± 21.7	100.7 ± 15.0	103.2 ± 10.1	106.6 ± 11.0	2.5 ± 11.1	5.9 ± 11.3 †
	Non	101.1 ± 12.8	108.4 ± 11.4	109.2 ± 9.4	103.0 ± 16.9	0.8 ± 6.2	-5.4 ± 8.2
Reaction time	Res	89.9 ± 20.6	95.5 ± 10.8	99.8 ± 12.8	99.1 ± 8.9	4.3 ± 14.4	3.6 ± 6.5
	Non	86.2 ± 18.7	92.7 ± 9.0	112.1 ± 29.6	89.7 ± 13.6	19.4 ± 34.2	-3.0 ± 10.8
Complex attention	Res	98.0 ± 20.6	103.9 ± 12.9	106.6 ± 11.5	111.4 ± 6.9*	2.7 ± 6.0	7.5 ± 8.9
	Non	77.5 ± 36.5	105.2 ± 10.2	65.1 ± 75.3	79.9 ± 63.0	-40.1 ± 72.2	-25.3 ± 59.7
Cognitive flexibility	Res	95.9 ± 8.0	98.2 ± 11.2	102.2 ± 9.5*	106.8 ± 8.6*	4.0 ± 4.7 †	8.6 ± 7.2 †
	Non	81.7 ± 19.0	101.8 ± 9.4	97.2 ± 13.0	96.0 ± 14.2	-4.6 ± 8.5	-5.8 ± 13.8
Processing speed	Res	103.6 ± 13.3	108.0 ± 11.6	109.2 ± 10.1	114.2 ± 8.7*	1.2 ± 9.5	6.2 ± 8.5 †
	Non	105.9 ± 11.1	110.3 ± 10.6	110.1 ± 9.1	105.0 ± 15.4	-0.2 ± 6.8	-5.3 ± 10.0
Executive function	Res	95.3 ± 7.8	98.4 ± 11.3	102.8 ± 8.0*	106.3 ± 8.6*	4.4 ± 5.4	7.9 ± 7.5 †
	Non	85.8 ± 12.3	102.3 ± 7.8	102.3 ± 6.8	97.6 ± 14.7	0 ± 5.6	-4.6 ± 16.5
Simple attention	Res	68.4 ± 122.7	99.4 ± 15.5	105.2 ± 5.4	103.6 ± 8.3	5.8 ± 14.3	4.1 ± 15.6
	Non	70.7 ± 70.7	98.7 ± 14.0	-63.4 ± 375.9	-1.7 ± 305.7	-162.1 ± 374.4	-100.5 ± 293.8
Motor speed	Res	90.9 ± 26.7	96.3 ± 15.8	98.8 ± 10.7	99.7 ± 10.3	2.6 ± 12.6	3.4 ± 12.1
	Non	97.7 ± 15.7	104.4 ± 12.3	105.5 ± 11.8	100.3 ± 14.9	1.2 ± 6.4	-4.1 ± 7.3

Res and Non denote the responder and non-responder subgroups, respectively. BW4: four weeks before food intervention; W0: just before intervention; W4: four weeks after intervention; W8: eight weeks after intervention. Paired t-tests were performed for within-group comparisons (W0 vs. W4 and W0 vs. W8) (*, indicates p-value < 0.05, after correction by the Benjamini-Hochberg method). Δ value represents the change in score from the baseline W0, and an unpaired t-test was performed between the responders and non-responders (†, p-value less than 0.05).

Supplementary Table S6. Cognitive domain scores of the Cognitrax test in the female Rice bran group.

Cognitive domain	subgroup	Score (mean ± SD)				Δ value	
		BW4	W0	W4	W8	W4	W8
Neurocognitive index (NCI)	Res	94.0 ± 13.0	97.1 ± 11.0	102.8 ± 9.0*	105.9 ± 6.1*	5.8 ± 6.0	8.8 ± 9.4 †
	Non	98.0 ± 13.6	105.9 ± 2.7	103.4 ± 8.7	103.3 ± 3.1	-2.4 ± 8.8	-2.6 ± 4.1
Composite memory	Res	83.4 ± 19.3	84.9 ± 16.5	94.4 ± 15.2*	97.4 ± 12.5*	9.5 ± 16.5	12.5 ± 16.7 †
	Non	100.7 ± 14.8	106.4 ± 15.5	107.9 ± 12.0	95.0 ± 20.9	1.4 ± 12.3	-11.4 ± 18.7
Verbal memory	Res	80.9 ± 22.5	87.2 ± 23.2	100.8 ± 19.4*	106.6 ± 16.0*	13.6 ± 21.4	19.4 ± 24.5 †
	Non	103.1 ± 17.5	106.6 ± 14.5	109.4 ± 19.1	99.3 ± 21.8	2.9 ± 16.8	-7.3 ± 21.0
Visual memory	Res	91.6 ± 13.8	87.8 ± 9.6	90.3 ± 16.3	89.6 ± 11.1	2.5 ± 19.5	1.8 ± 12.7 †
	Non	99.3 ± 10.2	104.7 ± 12.3	104.6 ± 11.4	92.6 ± 13.8	-0.1 ± 14.0	-12.1 ± 11.6
Psychomotor speed	Res	98.9 ± 15.0	105.0 ± 12.8	108.9 ± 10.3*	110.3 ± 12.2	3.9 ± 6.8	5.3 ± 11.5
	Non	104.0 ± 9.4	107.3 ± 8.1	108.7 ± 10.0	107.0 ± 8.7	1.4 ± 3.8	-0.3 ± 5.3
Reaction time	Res	93.2 ± 14.9	93.8 ± 9.8	95.6 ± 12.0	98.1 ± 8.0	1.8 ± 5.3	4.3 ± 6.2
	Non	95.9 ± 7.9	98.6 ± 9.2	98.0 ± 6.8	99.1 ± 6.6	-0.6 ± 5.8	0.6 ± 5.8
Complex attention	Res	97.9 ± 21.1	102.3 ± 19.3	109.8 ± 9.0	114.0 ± 5.9*	7.5 ± 15.2	11.7 ± 18.9 †
	Non	85.3 ± 66.3	110.7 ± 3.1	94.1 ± 42.2	107.9 ± 7.0	-16.6 ± 43.1	-2.9 ± 5.1
Cognitive flexibility	Res	96.6 ± 16.7	98.5 ± 15.0	105.1 ± 11.4*	109.7 ± 7.7*	6.6 ± 8.3 †	11.2 ± 12.3 †
	Non	105.0 ± 4.1	106.6 ± 5.9	108.1 ± 5.5	107.0 ± 8.3	1.6 ± 2.2	0.4 ± 6.6
Processing speed	Res	107.7 ± 11.0	110.7 ± 10.5	115.4 ± 11.6*	115.6 ± 12.9	4.7 ± 8.1	4.9 ± 11.1
	Non	107.6 ± 13.0	114.4 ± 9.3	111.4 ± 10.3	114.1 ± 9.4	-3.0 ± 7.4	-0.3 ± 8.0
Executive function	Res	97.1 ± 15.9	97.9 ± 14.4	104.9 ± 11.4*	108.9 ± 7.6*	6.9 ± 7.5 †	10.9 ± 11.5 †
	Non	104.3 ± 4.4	106.3 ± 6.0	107.9 ± 5.9	107.1 ± 8.4	1.6 ± 1.6	0.9 ± 7.0
Simple attention	Res	84.9 ± 42.6	89.7 ± 35.9	102.4 ± 8.4	104.8 ± 6.7	12.7 ± 34.9	15.1 ± 35.7
	Non	-62.1 ± 419.2	106.6 ± 7.3	-2.7 ± 265.9	105.1 ± 7.3	-109.3 ± 267.7	-1.4 ± 3.5
Motor speed	Res	94.0 ± 16.9	100.1 ± 16.2	102.6 ± 12.3	104.0 ± 13.8	2.5 ± 7.2	3.9 ± 11.1
	Non	100.3 ± 9.9	100.4 ± 9.0	102.9 ± 8.8	99.3 ± 9.1	2.4 ± 4.3	-1.1 ± 4.9

Res and Non denote the responder and non-responder subgroups, respectively. BW4: four weeks before food intervention; W0: just before intervention; W4: four weeks after intervention; W8: eight weeks after intervention. Paired t-tests were performed for within-group comparisons (W0 vs. W4 and W0 vs. W8) (*, indicates *p*-value < 0.05, after correction by the Benjamini-Hochberg method). Δ value represents the change in score from the baseline W0, and an unpaired t-test was performed between the responders and non-responders (†, *p*-value less than 0.05).

Supplementary Table S7. Relative abundance values and detection rates of intestinal bacteria that varied after intervention in the male Tamogitake responder subgroup. A Friedman test was performed on the central log-ratio-transformed abundances at W0 (just before intervention), W4 (four weeks after intervention), and W8 (eight weeks after intervention) for each of the intestinal bacterial taxa (genus level), and the taxa with significant differences (p-value < 0.05) were defined as those that varied after the test food intervention. Only intestinal bacteria with a detection rate of 25% or more in this subgroup at W0 are shown.

Taxa (genus level)	Relative abundance (mean ± SD, %)				Detection rate (%)			
	BW4	W0	W4	W8	BW4	W0	W4	W8
Agathobaculum	0.16 ± 0.16	0.15 ± 0.18	0.22 ± 0.19	0.11 ± 0.15	73.9	65.2	78.3	47.8
Anaerobutyricum	1.13 ± 0.79	1.19 ± 1.09	0.75 ± 0.69	1.01 ± 0.93	91.3	91.3	87.0	82.6
Blautia	4.65 ± 2.24	4.80 ± 2.25	3.61 ± 1.94	4.18 ± 2.36	100.0	100.0	100.0	100.0
Collinsella	3.63 ± 2.95	3.67 ± 2.66	4.69 ± 3.79	3.70 ± 3.31	87.0	87.0	87.0	87.0
Enterocloster	0.48 ± 1.05	0.28 ± 0.56	0.51 ± 1.09	0.75 ± 1.83	91.3	65.2	78.3	43.5
Erysipelatoclostridium	0.09 ± 0.12	0.08 ± 0.20	0.07 ± 0.10	0.10 ± 0.20	52.2	52.2	52.2	47.8
Faecalibacillus	0.42 ± 0.90	0.52 ± 0.99	0.29 ± 0.56	0.41 ± 0.73	30.4	34.8	34.8	34.8
Megamonas	1.60 ± 4.46	1.13 ± 1.98	0.74 ± 2.81	1.82 ± 5.29	34.8	39.1	30.4	39.1
Parabacteroides	3.41 ± 2.62	3.14 ± 2.59	2.86 ± 2.85	3.78 ± 3.72	95.7	95.7	91.3	91.3
Phascolarctobacterium	0.31 ± 0.58	0.44 ± 0.70	0.28 ± 0.55	0.35 ± 0.62	52.2	56.5	52.2	60.9
*Ruminococcus*2	0.69 ± 0.88	0.70 ± 0.79	0.64 ± 1.77	0.27 ± 0.44	82.6	87.0	73.9	56.5

BW4: Four weeks before the intervention.

Supplementary Table S8. Relative abundance values and detection rates of intestinal bacteria that varied after intervention in the male Tamogitake non-responder subgroup. A Friedman test was performed on the central log-ratio-transformed abundances at W0 (just before intervention), W4 (four weeks after intervention), and W8 (eight weeks after intervention) for each of the intestinal bacterial taxa (genus level), and the taxa with significant differences (*p*-value < 0.05) were defined as those that varied after the test food intervention. Only intestinal bacteria with a detection rate of 25% or more in this subgroup at W0 are shown.

Taxa (genus level)	Relative abundance (mean ± SD, %)				Detection rate (%)			
	BW4	W0	W4	W8	BW4	W0	W4	W8
Agathobacter	1.34 ± 2.04	1.42 ± 2.38	0.28 ± 0.55	2.41 ± 3.27	53.8	53.8	46.2	53.8
Alistipes	0.62 ± 0.58	0.65 ± 0.57	0.83 ± 1.13	0.40 ± 0.37	84.6	76.9	84.6	84.6
Allisonella	0.04 ± 0.05	0.03 ± 0.04	0.05 ± 0.08	0.06 ± 0.07	46.2	46.2	46.2	53.8
Holdemanella	1.74 ± 2.37	1.28 ± 1.78	0.50 ± 0.74	1.46 ± 2.19	53.8	53.8	53.8	53.8
*Ruminococcus*2	0.75 ± 0.96	0.63 ± 0.83	0.26 ± 0.49	0.34 ± 0.45	69.2	69.2	61.5	61.5
Unclassified	13.65 ± 9.20	13.46 ± 7.77	13.93 ± 8.32	10.73 ± 6.14	100.0	100.0	100.0	100.0

BW4: Four weeks before the intervention.

Supplementary Table S9. Relative abundance values and detection rates of intestinal bacteria that varied after intervention in the female Tamogitake responder subgroup. A Friedman test was performed on the central log-ratio-transformed abundances at W0 (just before intervention), W4 (four weeks after intervention), and W8 (eight weeks after intervention) for each of the intestinal bacterial taxa (genus level), and the taxa with significant differences (p-value < 0.05) were defined as those that varied after the test food intervention. Only intestinal bacteria with a detection rate of 25% or more in this subgroup at W0 are shown.

Taxa (genus level)	Relative abundance (mean ± SD, %)				Detection rate (%)			
	BW4	W0	W4	W8	BW4	W0	W4	W8
Agathobaculum	0.26 ± 0.26	0.21 ± 0.18	0.43 ± 0.45	0.25 ± 0.28	89.5	84.2	84.2	84.2
Blautia	5.99 ± 2.02	5.10 ± 2.29	3.03 ± 2.24	5.93 ± 3.76	100.0	94.7	100.0	100.0
Collinsella	3.29 ± 3.36	3.36 ± 3.19	5.14 ± 5.61	3.65 ± 3.52	68.4	68.4	68.4	73.7
Dorea	0.82 ± 0.77	1.08 ± 1.13	0.44 ± 0.61	0.82 ± 0.69	68.4	68.4	57.9	78.9
Enterocloster	0.41 ± 0.57	0.41 ± 0.56	0.41 ± 0.60	0.29 ± 0.48	94.7	68.4	73.7	57.9
Lawsonibacter	0.11 ± 0.14	0.09 ± 0.10	0.17 ± 0.20	0.10 ± 0.14	68.4	52.6	57.9	52.6
Massilimicrobiota	0.08 ± 0.13	0.04 ± 0.07	0.01 ± 0.04	0.05 ± 0.08	52.6	31.6	15.8	52.6
Parabacteroides	2.44 ± 4.17	3.37 ± 5.86	2.18 ± 4.21	2.93 ± 3.48	84.2	84.2	84.2	84.2

BW4: Four weeks before the intervention.

Supplementary Table S10. Relative abundance values and detection rates of intestinal bacteria that varied after intervention in the female Tamogitake non-responder subgroup. A Friedman test was performed on the central log-ratio-transformed abundances at W0 (just before intervention), W4 (four weeks after intervention), and W8 (eight weeks after intervention) for each of the intestinal bacterial taxa (genus level), and the taxa with significant differences (p-value < 0.05) were defined as those that varied after the test food intervention. Only intestinal bacteria with a detection rate of 25% or more in this subgroup at W0 are shown.

Taxa (genus level)	Relative abundance (mean ± SD, %)				Detection rate (%)			
	BW4	W0	W4	W8	BW4	W0	W4	W8
Dielma	0.01 ± 0.01	0.01 ± 0.01	0.01 ± 0.02	0.01 ± 0.02	25.0	25.0	25.0	25.0
Duodenibacillus	0.12 ± 0.22	0.03 ± 0.06	0.07 ± 0.12	0.14 ± 0.25	25.0	25.0	25.0	25.0
Dysosmobacter	0.51 ± 0.24	0.61 ± 0.19	0.34 ± 0.22	0.46 ± 0.34	100.0	100.0	100.0	100.0
Fusobacterium	0.08 ± 0.11	0.01 ± 0.01	0.08 ± 0.13	0.04 ± 0.05	50.0	50.0	50.0	50.0
Lachnospira	1.72 ± 1.75	0.44 ± 0.36	1.27 ± 0.78	0.44 ± 0.44	75.0	75.0	75.0	50.0
Longibaculum	0.00 ± 0.01	0.01 ± 0.02	0.00 ± 0.00	0.32 ± 0.55	25.0	25.0	0.0	25.0
Longicatena	0.34 ± 0.49	0.28 ± 0.49	0.43 ± 0.75	0.37 ± 0.55	50.0	25.0	25.0	50.0
Megasphaera	0.19 ± 0.34	0.09 ± 0.16	0.14 ± 0.24	0.12 ± 0.20	25.0	25.0	25.0	25.0
Peptacetobacter	0.76 ± 1.31	0.10 ± 0.18	0.18 ± 0.32	0.21 ± 0.37	25.0	25.0	25.0	25.0
Phascolarctobacterium	0.24 ± 0.41	0.26 ± 0.27	0.30 ± 0.33	0.69 ± 0.76	50.0	50.0	50.0	50.0
Sellimonas	0.06 ± 0.10	0.04 ± 0.07	0.02 ± 0.04	0.05 ± 0.09	25.0	25.0	25.0	25.0

BW4: Four weeks before the intervention.

Supplementary Table S11. Relative abundance values and detection rates of intestinal bacteria that varied after intervention in the male Moringa responder subgroup. A Friedman test was performed on the central log-ratio-transformed abundances at W0 (just before intervention), W4 (four weeks after intervention), and W8 (eight weeks after intervention) for each of the intestinal bacterial taxa (genus level), and the taxa with significant differences (*p*-value < 0.05) were defined as those that varied after the test food intervention. Only intestinal bacteria with a detection rate of 25% or more in this subgroup at W0 are shown.

Taxa (genus level)	Relative abundance (mean ± SD, %)				Detection rate (%)			
	BW4	W0	W4	W8	BW4	W0	W4	W8
Agathobaculum	0.36 ± 0.61	0.21 ± 0.34	0.42 ± 0.67	0.16 ± 0.26	64.0	64.0	88.0	60.0
Anaerobutyricum	0.91 ± 0.98	1.00 ± 1.01	0.67 ± 1.41	0.67 ± 0.76	84.0	84.0	84.0	92.0
Anaerostipes	0.55 ± 0.80	0.60 ± 0.78	0.36 ± 0.53	0.50 ± 0.53	84.0	84.0	68.0	76.0
Bacteroides	8.01 ± 11.12	7.41 ± 9.51	6.16 ± 7.93	7.48 ± 7.56	100.0	100.0	96.0	100.0
Butyricicoccus	0.10 ± 0.24	0.04 ± 0.08	0.15 ± 0.31	0.08 ± 0.23	24.0	28.0	40.0	16.0
Dysosmobacter	0.28 ± 0.40	0.32 ± 0.32	0.57 ± 0.64	0.23 ± 0.23	88.0	64.0	80.0	72.0
Enterocloster	0.18 ± 0.23	0.13 ± 0.25	0.13 ± 0.16	0.13 ± 0.19	84.0	52.0	60.0	56.0
Intestinimonas	0.05 ± 0.14	0.04 ± 0.06	0.11 ± 0.17	0.05 ± 0.08	40.0	40.0	52.0	40.0
Phascolarctobacterium	0.57 ± 0.93	0.71 ± 1.37	0.53 ± 0.93	0.69 ± 1.08	64.0	72.0	60.0	72.0
Unclassified	13.76 ± 6.67	12.84 ± 5.11	15.26 ± 8.82	13.85 ± 6.42	100.0	100.0	100.0	100.0

BW4: Four weeks before the intervention.

Supplementary Table S12. Relative abundance values and detection rates of intestinal bacteria that varied after intervention in the male Moringa non-responder subgroup. A Friedman test was performed on the central log-ratio-transformed abundances at W0 (just before intervention), W4 (four weeks after intervention), and W8 (eight weeks after intervention) for each of the intestinal bacterial taxa (genus level), and the taxa with significant differences (p-value < 0.05) were defined as those that varied after the test food intervention. Only intestinal bacteria with a detection rate of 25% or more in this subgroup at W0 are shown.

Taxa (genus level)	Relative abundance (mean ± SD, %)				Detection rate (%)			
	BW4	W0	W4	W8	BW4	W0	W4	W8
Acidaminococcus	0.16 ± 0.34	0.18 ± 0.42	0.39 ± 0.98	0.07 ± 0.11	27.3	36.4	36.4	36.4
Catenibacterium	0.93 ± 1.42	1.14 ± 1.97	1.47 ± 2.21	1.01 ± 1.72	36.4	36.4	36.4	36.4
Coprococcus	0.14 ± 0.14	0.30 ± 0.33	0.13 ± 0.20	0.34 ± 0.31	63.6	54.5	36.4	72.7
Mediterraneibacter	0.37 ± 0.30	0.30 ± 0.39	0.10 ± 0.12	0.62 ± 0.90	90.9	72.7	54.5	81.8
Megasphaera	0.90 ± 1.15	0.47 ± 0.91	0.34 ± 0.43	0.44 ± 0.97	54.5	45.5	45.5	36.4
Romboutsia	0.21 ± 0.24	0.32 ± 0.53	0.22 ± 0.50	0.21 ± 0.22	72.7	72.7	18.2	72.7
Roseburia	0.86 ± 0.94	0.92 ± 1.26	0.74 ± 1.42	1.36 ± 1.49	72.7	81.8	72.7	81.8
Veillonella	0.07 ± 0.13	0.24 ± 0.58	0.48 ± 1.25	0.29 ± 0.60	36.4	36.4	27.3	36.4

BW4: Four weeks before the intervention.

Supplementary Table S13. Relative abundance values and detection rates of intestinal bacteria that varied after intervention in the female Moringa responder subgroup. A Friedman test was performed on the central log-ratio-transformed abundances at W0 (just before intervention), W4 (four weeks after intervention), and W8 (eight weeks after intervention) for each of the intestinal bacterial taxa (genus level), and the taxa with significant differences (p-value < 0.05) were defined as those that varied after the test food intervention. Only intestinal bacteria with a detection rate of 25% or more in this subgroup at W0 are shown.

Taxa (genus level)	Relative abundance (mean ± SD, %)				Detection rate (%)			
	BW4	W0	W4	W8	BW4	W0	W4	W8
Adlercreutzia	0.10 ± 0.10	0.21 ± 0.27	0.21 ± 0.26	0.16 ± 0.27	75.0	62.5	75.0	56.3
Agathobaculum	0.17 ± 0.20	0.19 ± 0.34	0.30 ± 0.35	0.25 ± 0.40	68.8	56.3	81.3	75.0
*Clostridium*_IV	0.42 ± 0.99	0.26 ± 0.35	0.18 ± 0.34	0.20 ± 0.45	50.0	75.0	56.3	50.0
*Clostridium*_XVIII	0.04 ± 0.06	0.10 ± 0.17	0.03 ± 0.05	0.15 ± 0.35	37.5	50.0	31.3	37.5
Collinsella	2.88 ± 3.22	3.60 ± 3.83	3.63 ± 3.57	2.56 ± 2.15	68.8	68.8	68.8	68.8
Faecalibacterium	9.16 ± 7.95	8.97 ± 8.11	12.88 ± 10.16	10.64 ± 7.59	87.5	87.5	93.8	100.0
Intestinimonas	0.09 ± 0.10	0.12 ± 0.27	0.13 ± 0.17	0.12 ± 0.18	68.8	56.3	62.5	43.8
Paraprevotella	0.30 ± 0.38	0.14 ± 0.20	0.46 ± 0.95	0.27 ± 0.44	43.8	43.8	43.8	43.8

BW4: Four weeks before the intervention.

Supplementary Table S14. Relative abundance values and detection rates of intestinal bacteria that varied after intervention in the female Moringa non-responder subgroup. A Friedman test was performed on the central log-ratio-transformed abundances at W0 (just before intervention), W4 (four weeks after intervention), and W8 (eight weeks after intervention) for each of the intestinal bacterial taxa (genus level), and the taxa with significant differences (*p*-value < 0.05) were defined as those that varied after the test food intervention. Only intestinal bacteria with a detection rate of 25% or more in this subgroup at W0 are shown.

Taxa (genus level)	Relative abundance (mean ± SD, %)				Detection rate (%)			
	BW4	W0	W4	W8	BW4	W0	W4	W8
Anaerotignum	0.25 ± 0.15	0.08 ± 0.06	0.05 ± 0.05	0.44 ± 0.30	100.0	75.0	50.0	100.0
Coprococcus	0.57 ± 0.50	0.35 ± 0.33	0.16 ± 0.11	0.44 ± 0.41	75.0	75.0	75.0	75.0
Fusobacterium	0.12 ± 0.21	0.09 ± 0.15	0.15 ± 0.26	0.13 ± 0.22	25.0	25.0	25.0	25.0
Lacticaseibacillus	0.32 ± 0.55	0.02 ± 0.03	0.70 ± 1.21	0.41 ± 0.70	25.0	25.0	25.0	25.0
Mediterraneibacter	0.83 ± 0.50	0.95 ± 0.61	0.29 ± 0.51	1.37 ± 1.10	75.0	75.0	25.0	75.0
Parasutterella	0.27 ± 0.47	0.08 ± 0.14	0.12 ± 0.21	0.18 ± 0.31	25.0	25.0	25.0	25.0

BW4: Four weeks before the intervention.

Supplementary Table S15. Relative abundance values and detection rates of intestinal bacteria that varied after intervention in the male Rice bran responder subgroup. A Friedman test was performed on the central log-ratio-transformed abundances at W0 (just before intervention), W4 (four weeks after intervention), and W8 (eight weeks after intervention) for each of the intestinal bacterial taxa (genus level), and the taxa with significant differences (p-value < 0.05) were defined as those that varied after the test food intervention. Only intestinal bacteria with a detection rate of 25% or more in this subgroup at W0 are shown.

Taxa (genus level)	Relative abundance (mean ± SD, %)				Detection rate (%)			
	BW4	W0	W4	W8	BW4	W0	W4	W8
Adlercreutzia	0.04 ± 0.05	0.04 ± 0.07	0.10 ± 0.18	0.13 ± 0.19	44.4	38.9	61.1	50.0
Anaerobutyricum	0.77 ± 0.55	0.92 ± 0.77	0.47 ± 0.39	0.94 ± 0.68	83.3	77.8	83.3	83.3
Blautia	5.99 ± 4.31	5.78 ± 4.50	3.90 ± 4.09	5.92 ± 5.67	100.0	94.4	94.4	100.0
Faecalibacterium	6.38 ± 5.67	6.79 ± 6.53	12.89 ± 13.57	6.72 ± 6.71	77.8	72.2	72.2	72.2
Holdemania	0.06 ± 0.10	0.11 ± 0.19	0.13 ± 0.26	0.07 ± 0.10	61.1	66.7	83.3	55.6
Parabacteroides	2.23 ± 1.90	2.51 ± 1.94	1.69 ± 1.54	2.40 ± 2.30	94.4	94.4	94.4	88.9
*Ruminococcus*2	0.84 ± 0.80	0.80 ± 0.83	0.41 ± 0.50	0.57 ± 0.74	77.8	77.8	72.2	66.7
Unclassified	16.55 ± 8.40	15.88 ± 7.97	18.82 ± 9.74	16.54 ± 9.44	100.0	100.0	100.0	100.0

BW4: Four weeks before the intervention.

Supplementary Table S16. Relative abundance values and detection rates of intestinal bacteria that varied after intervention in the male Rice bran non-responder subgroup. A Friedman test was performed on the central log-ratio-transformed abundances at W0 (just before intervention), W4 (four weeks after intervention), and W8 (eight weeks after intervention) for each of the intestinal bacterial taxa (genus level), and the taxa with significant differences (*p*-value < 0.05) were defined as those that varied after the test food intervention. Only intestinal bacteria with a detection rate of 25% or more in this subgroup at W0 are shown.

Taxa (genus level)	Relative abundance (mean ± SD, %)				Detection rate (%)			
	BW4	W0	W4	W8	BW4	W0	W4	W8
Anaerostipes	0.68 ± 1.03	0.67 ± 0.87	0.26 ± 0.37	0.37 ± 0.31	90.9	81.8	72.7	81.8
Anaerotignum	0.27 ± 0.25	0.27 ± 0.27	0.08 ± 0.10	0.31 ± 0.29	100.0	100.0	72.7	90.9
Collinsella	3.26 ± 1.64	3.72 ± 2.07	4.08 ± 2.27	3.02 ± 2.50	90.9	90.9	90.9	90.9
Erysipelatoclostridium	0.15 ± 0.25	0.04 ± 0.07	0.06 ± 0.10	0.11 ± 0.24	45.5	36.4	45.5	36.4
Parabacteroides	2.66 ± 2.95	3.29 ± 3.25	2.38 ± 2.83	4.21 ± 5.99	81.8	81.8	81.8	81.8
Sellimonas	0.01 ± 0.02	0.01 ± 0.02	0.07 ± 0.14	0.05 ± 0.12	18.2	27.3	27.3	18.2
Streptococcus	2.15 ± 5.06	1.94 ± 3.99	0.68 ± 1.40	1.27 ± 3.83	81.8	81.8	81.8	45.5
Turicibacter	0.10 ± 0.21	0.21 ± 0.50	0.54 ± 1.44	0.14 ± 0.34	36.4	36.4	54.5	36.4

BW4: Four weeks before the intervention.

Supplementary Table S17. Relative abundance values and detection rates of intestinal bacteria that varied after intervention in the female Rice bran responder subgroup. A Friedman test was performed on the central log-ratio-transformed abundances at W0 (just before intervention), W4 (four weeks after intervention), and W8 (eight weeks after intervention) for each of the intestinal bacterial taxa (genus level), and the taxa with significant differences (*p*-value < 0.05) were defined as those that varied after the test food intervention. Only intestinal bacteria with a detection rate of 25% or more in this subgroup at W0 are shown.

**Taxa (genus level)**	**Relative abundance (mean ± SD, %)**				**Detection rate (%)**			
	**BW4**	**W0**	**W4**	**W8**	**BW4**	**W0**	**W4**	**W8**
Bifidobacterium	8.49 ± 8.60	8.03 ± 7.15	7.89 ± 7.22	5.31 ± 5.76	89.5	89.5	100.0	84.2
Faecalibacterium	8.26 ± 6.40	8.31 ± 6.76	13.03 ± 9.49	10.73 ± 7.36	89.5	89.5	100.0	100.0
Parabacteroides	1.55 ± 1.12	1.84 ± 1.53	1.57 ± 1.66	1.75 ± 1.38	84.2	84.2	84.2	84.2
Unclassified	17.75 ± 8.02	16.90 ± 6.26	17.02 ± 7.61	14.60 ± 6.75	100.0	100.0	100.0	100.0

BW4: Four weeks before the intervention.

Supplementary Table S18. Relative abundance values and detection rates of intestinal bacteria that varied after intervention in the female Rice bran non-responder subgroup. A Friedman test was performed on the central log-ratio-transformed abundances at W0 (just before intervention), W4 (four weeks after intervention), and W8 (eight weeks after intervention) for each of the intestinal bacterial taxa (genus level), and the taxa with significant differences (p-value < 0.05) were defined as those that varied after the test food intervention. Only intestinal bacteria with a detection rate of 25% or more in this subgroup at W0 are shown.

Taxa (genus level)	Relative abundance (mean ± SD, %)				Detection rate (%)			
	BW4	W0	W4	W8	BW4	W0	W4	W8
Adlercreutzia	0.05 ± 0.11	0.05 ± 0.07	0.13 ± 0.18	0.08 ± 0.12	28.6	42.9	42.9	42.9
Akkermansia	0.19 ± 0.43	0.21 ± 0.34	0.18 ± 0.43	0.33 ± 0.46	28.6	28.6	28.6	42.9
Anaeromassilibacillus	0.03 ± 0.02	0.08 ± 0.08	0.03 ± 0.05	0.01 ± 0.02	71.4	71.4	57.1	42.9
Bacteroides	8.95 ± 6.61	11.75 ± 6.85	10.78 ± 7.74	10.18 ± 7.51	100.0	100.0	100.0	100.0
*Clostridium*_XlVb	0.20 ± 0.32	0.22 ± 0.38	0.08 ± 0.15	0.36 ± 0.52	28.6	28.6	28.6	42.9
Flavonifractor	0.21 ± 0.23	0.36 ± 0.35	0.45 ± 0.62	0.20 ± 0.23	57.1	71.4	85.7	57.1
Intestinimonas	0.17 ± 0.38	0.12 ± 0.19	0.35 ± 0.49	0.19 ± 0.22	42.9	42.9	57.1	57.1
Mediterraneibacter	0.11 ± 0.19	0.07 ± 0.13	0.07 ± 0.16	0.03 ± 0.08	57.1	28.6	28.6	14.3
Odoribacter	0.28 ± 0.27	0.33 ± 0.11	0.15 ± 0.11	0.27 ± 0.24	100.0	100.0	100.0	100.0

BW4: Four weeks before the intervention.

Supplementary Table S19. Health status of participants included in analysis. The number of participants free from disease and with disease in each subgroup are shown.

	Tamogitake group				Moringa group				Rice bran group			
	Male		Female		Male		Female		Male		Female	
	Res (23)	Non (13)	Res (19)	Non (4)	Res (25)	Non (11)	Res (16)	Non (4)	Res (18)	Non (11)	Res (19)	Non (7)
Free from disease	6	4	9	2	13	5	9	3	7	8	12	5
Disease:												
Type 2 diabetes	4	2	1	0	1	1	0	0	2	0	0	0
Dyslipidemia	2	0	1	1	1	1	1	0	2	0	1	0
Hypertension	8	6	3	1	5	3	1	0	4	2	1	1
Angina pectoris	0	0	0	0	1	0	0	0	0	0	0	0
Arrhythmia	0	1	0	0	0	0	0	0	0	0	0	0
Cerebral infarction	1	0	0	0	0	0	0	0	0	0	0	0
Diseases of circulatory system	1	0	0	0	0	0	0	0	0	0	0	0
Constipation	0	0	0	0	0	0	0	0	0	1	0	0
Hemorrhoids	0	0	0	0	0	0	1	0	0	0	0	0
Chronic kidney disease	0	0	0	0	1	0	0	0	0	0	0	0
Benign prostatic hyperplasia	0	0	0	0	0	2	0	0	1	0	0	0
Asthma	1	0	0	1	0	0	0	0	0	0	1	0
Pollinosis	1	2	3	0	1	1	1	0	2	1	0	0
Allergies	0	0	1	0	0	1	0	0	0	0	0	0
Respiratory or otolaryngological diseases	1	0	1	0	0	0	0	1	0	0	0	0
Diseases of skin and subcutaneous tissue	0	1	0	0	1	0	0	0	0	0	0	0
Rheumatoid arthritis	0	0	0	0	0	0	1	0	0	0	0	0
Gout	2	1	0	0	1	1	0	0	0	1	0	0
Osteoporosis	0	0	1	0	0	0	0	0	0	0	0	0
Hip osteoarthritis	0	0	0	1	0	0	1	0	0	0	1	0
Spinal stenosis	0	0	0	1	0	0	0	0	0	0	0	0
Low back pain	1	0	0	0	0	0	0	0	1	0	0	0
Cataract	1	0	0	0	0	0	0	0	1	1	0	0
Glaucoma	1	0	0	1	1	0	0	0	2	1	0	0
Depression	0	0	0	0	1	0	0	0	0	0	0	0
Sleep apnea syndrome	1	0	0	0	0	0	0	0	0	0	0	0
Obesity	1	0	0	0	0	0	0	0	1	0	0	0
Periodontal disease	1	0	0	0	1	0	0	0	0	1	0	1
Headache, migraine	1	0	0	0	0	0	1	0	0	0	0	0
Anemia	0	0	0	0	0	0	0	0	1	0	0	0
Sensitivity to cold	1	0	0	0	1	0	0	1	1	0	1	0
Malaise and fatigue	0	0	0	1	0	0	0	0	1	0	1	0
Other diseases	1	0	0	0	0	1	1	1	0	0	0	0

Res and Non denote responder and non-responder subgroups, respectively. Numbers in parentheses show the total number of participants in the subgroup. Some participants had multiple diseases.
